# Supplementary material for: Sensitivity to visual features in inattentional blindness
Source: eLife. 2025 May 19;13:RP100337. doi: 10.7554/eLife.100337 (PMC12088676; doi:10.7554/eLife.100337)
Supplement: Peer review history

# Editor report: eLife Assessment

**DOI:** 10.7554/eLife.100337.3.sa0

**Authors:** Simon van Gaal

**Affiliations:** University of Amsterdam Netherlands

**Keywords:** Convincing, Valuable

This study presents **valuable** findings to the field interested in inattentional blindness (IB), the phenomenon that participants fail to notice salient stimuli when their attention is directed elsewhere. This study reveals that participants who indicate no awareness of unexpected stimuli through yes/no questions ("did you notice anything unusual?"), may still show above-chance sensitivity to specific properties of these stimuli through follow-up forced-choice questions (e.g., regarding its location or color). By introducing absent trials where no IB stimulus is presented, the authors show that this is because participants are generally conservative and biased to report not noticing in inattentional blindness experiments. The evidence supporting these conclusions is **convincing**, the samples sizes are large and the analysis protocol is novel.

# Referee report: Reviewer #1 (Public review)

**DOI:** 10.7554/eLife.100337.3.sa1

The results of these experiments support a modest but important conclusion: If sub-optimal methods are used to collect retrospective reports, such as simple yes/no questions, inattentional blindness (IB) rates may be overestimated by up to ~8%.

(1) In experiment 1, data from 374 subjects were included in the analysis. As shown in figure 2b, 267 subjects reported noticing the critical stimulus and 107 subjects reported not noticing it. This translates to a 29% IB rate if we were to only consider the "did you notice anything unusual Y/N" question. As reported in the results text (and figure 2c), when asked to report the location of the critical stimulus (left/right), 63.6% of the "non-noticer" group answered correctly. In other words, 68 subjects were correct about the location while 39 subjects were incorrect. Importantly, because the location judgment was a 2-alternative-forced-choice, the assumption was that if 50% (or at least not statistically different than 50%) of the subjects answered the location question correctly, everyone was purely guessing. Therefore, we can estimate that ~39 of the subjects who answered correctly were simply guessing (because 39 guessed incorrectly), leaving 29 subjects from the non-noticer group who were correct on the 2AFC above and beyond the pure guess rate. If these 29 subjects are moved from the non-noticer to the noticer group, the corrected rate of IB for Experiment 1 is 20.86% instead of the original 28.61% rate that would have been obtained if only the Y/N question was used. In other words, relying only on the "Y/N did you notice anything" question led to an overestimate of IB rates by 7.75% in Experiment 1.

In the revised version of their manuscript, the authors provided the data that was missing from the original submission, which allows this same exercise to be carried out on the other 4 experiments. Using the same logic as above, i.e., calculating the pure-guess rate on the 2AFC, moving the number of subjects above this pure-guess rate to the non-noticer group, and then re-calculating a "corrected IB rate", the other experiments demonstrate the following:

Experiment 2: IB rates were overestimated by 4.74% (original IB rate based only on Y/N question = 27.73%; corrected IB rate that includes the 2AFC = 22.99%)

Experiment 3: IB rates were overestimated by 3.58% (original IB rate = 30.85%; corrected IB rate = 27.27%)

Experiment 4: IB rates were overestimated by ~8.19% (original IB rate = 57.32%; corrected IB rate for color* = 39.71%, corrected IB rate for shape = 52.61%, corrected IB rate for location = 55.07%)

Experiment 5: IB rates were overestimated by ~1.44% (original IB rate = 28.99%; corrected IB rate for color = 27.56%, corrected IB rate for shape = 26.43%, corrected IB rate for location = 28.65%)

*note: the highest overestimate of IB rates was from Experiment 4, color condition, but the authors admitted that there was a problem with 2AFC color guessing bias in this version of the experiment which was a main motivation for running experiment 5 which corrected for this bias.

Taken as a whole, this data clearly demonstrates that even with a conservative approach to analyzing the combination of Y/N and 2AFC data, inattentional blindness was evident in a sizeable portion of the subject populations. An important (albeit modest) overestimate of IB rates was demonstrated by incorporating these improved methods.

(2) One of the strongest pieces of evidence presented in this paper was the single data point in Figure 3e showing that in Experiment 3, even the super subject group that rated their non-noticing as "highly confident" had a d' score significantly above zero. Asking for confidence ratings is certainly an improvement over simple Y/N questions about noticing, and if this result were to hold, it could provide a key challenge to IB. However, this result can most likely be explained by measurement error.

In their revised paper, the authors reported data that was missing from their original submission: the confidence ratings on the 2AFC judgments that followed the initial Y/N question. The most striking indication that this data is likely due to measurement error comes from the number of subjects who indicated that they were highly confident that they didn't notice anything on the critical trial, but then when asked to guess the location of the stimulus, indicated that they were highly confident that the stimulus was on the left (or right). There were 18 subjects (8.82% of the high-confidence non-noticer group) who responded this way. To most readers, this combination of responses (high confidence in correctly judging a stimulus feature that one is highly confident in having not seen at all) indicates that a portion of subjects misunderstood the confidence scales (or just didn't read the questions carefully or made mistakes in their responses, which is common for experiments conducted online).

In the authors' rebuttal to the first round of peer review, they wrote, "it is perfectly rationally coherent to be very confident that one didn't see anything but also very confident that if there was anything to be seen, it was on the left." I respectfully disagree that such a combination of responses is rationally coherent. The more parsimonious interpretation is that a measurement error occurred, and it's questionable whether we should trust any responses from these 18 subjects.

In their rebuttal, the authors go on to note that 14 of the 18 subjects who rated their 2AFC with high confidence were correct in their location judgment. If these 14 subjects were removed from analysis (which seems like a reasonable analysis choice, given their contradictory responses), d' for the high-confidence non-noticer group would most likely fall to chance levels. In other words, we would see a data pattern similar to that plotted in Figure 3e, but with the first data point on the left moving down to zero d'. This corrected Figure 3e would then provide a very nice evidence-based justification for including confidence ratings along with Y/N questions in future inattentional blindness studies.

(3) In most (if not all) IB experiments in the literature, a partial attention and/or full attention trial is administered after the critical trial. These control trials are very important for validating IB on the critical trial, as they must show that, when attended, the critical stimuli are very easy to see. If a subject cannot detect the critical stimulus on the control trial, one cannot conclude that they were inattentionally blind on the critical trial, e.g., perhaps the stimulus was just too difficult to see (e.g., too weak, too brief, too far in the periphery, too crowded by distractor stimuli, etc.), or perhaps they weren't paying enough attention overall or failed to follow instructions. In the aggregate data, rates of noticing the stimuli should increase substantially from the critical trial to the control trials. If noticing rates are equivalent on the critical and control trials, one cannot conclude that attention was manipulated in the first place.

In their rebuttal to the first round of peer review, the authors provided weak justification for not including such a control condition. They cite one paper that argues such control conditions are often used to exclude subjects from analysis (those who fail to notice the stimulus on the control trial are either removed from analysis or replaced with new subjects) and such exclusions/replacements can lead to underestimations of inattentional blindness rates. However, the inclusion of a partial or full attention condition as a control does not necessitate the extra step of excluding or replacing subjects. In the broadest sense, such a control condition simply validates the attention manipulation, i.e., one can easily compare the percent of subjects who answered "yes" or who got the 2AFC judgment correct during the critical trial versus the control trial. The subsequent choice about exclusion/replacement is separate, and researchers can always report the data with and without such exclusions/replacements to remain more neutral on this practice.

If anyone were to follow-up on this study, I highly recommend including a partial or full attention control condition, especially given the online nature of data collection. It's important to know the percent of online subjects who answer yes and who get the 2AFC question correct when the critical stimulus is attended, because that is the baseline (in this case, the "ceiling level" of performance) to which the IB rates on the critical trial can be compared.

# Referee report: Reviewer #2 (Public review)

**DOI:** 10.7554/eLife.100337.3.sa2

In this study, Nartker et al. examine how much observers are conscious of using variations of classic inattentional blindness studies. The key idea is that rather than simply ask observers if they noticed a critical object with one yes/no question, the authors also ask follow-up questions to determine if observers are aware of more than the yes/no questions suggest. Specifically, by having observers make forced choice guesses about the critical object, the authors find that many observers who initially said "no" they did not see the object can still "guess" above chance about the critical object's location, color, etc. Thus, the authors claim, that prior claims of inattentional blindness are mistaken and that using such simple methods has led numerous researchers to overestimate how little observers see in the world. To quote the authors themselves, these results imply that "inattentionally blind subjects consciously perceive these stimuli after all... they show sensitivity to IB stimuli because they can see them."

Before getting to a few issues I have with the paper, I do want to make sure to explicitly compliment the researchers for many aspects of their work. Getting massive amounts of data, using signal detection measures, and the novel use of a "super subject" are all important contributions to the literature that I hope are employed more in the future.

Main point 1: My primary issue with this work is that I believe the authors are misrepresenting the way people often perform inattentional blindness studies. In effect, the authors are saying, "People do the studies 'incorrectly' and report that people see very little. We perform the studies 'correctly' and report that people see much more than previously thought." But the way previous studies are conducted is not accurately described in this paper. The authors describe previous studies as follows on page 3:

"Crucially, however, this interpretation of IB and the many implications that follow from it rest on a measure that psychophysics has long recognized to be problematic: simply asking participants whether they noticed anything unusual. In IB studies, awareness of the unexpected stimulus (the novel shape, the parading gorilla, etc.) is retroactively probed with a yes/no question, standardly, "Did you notice anything unusual on the last trial which wasn't there on previous trials?". Any subject who answers "no" is assumed not to have any awareness of the unexpected stimulus.

If this quote were true, the authors would have a point. Unfortunately, I do not believe it is true. This is simply not how many inattentional blindness studies are run. Some of the most famous studies in the inattentional blindness literature do not simply as observes a yes/no question e.g., the invisible gorilla (Simons et al. 1999), the classic door study where the person changes (Simons and Levin, 1998), the study where observers do not notice a fight happening a few feet from them (Chabris et al., 2011). Instead, these papers consistently ask a series of follow-up questions and even tell the observers what just occurred to confirm that observers did not notice that critical event (e.g., "If I were to tell you we just did XYZ, did you notice that?"). In fact, after a brief search on Google Scholar, I was able to relatively quickly find over a dozen papers that do not just use a yes/no procedure, and instead as a series of multiple questions to determine if someone is inattentionally blind. In no particular order some papers:

(1) Most et al. (2005) Psych Review
(2) Drew et al. (2013) Psych Science
(3) Drew et al. (2016) Journal of Vision
(4) Simons et al. (1999) Perception
(5) Simons and Levin (1998) Perception
(6) Chabris et al. (2011) iPerception
(7) Ward & Scholl (2015) Psych Bulletin and Review
(8) Most et al. (2001) Psych Science
(9) Todd & Marois (2005) Psych Science
(10) Fougnie & Marois (2007) Psych Bulletin and Review
(11) New and German (2015) Evolution and Human Behaviour
(12) Jackson-Nielsen (2017) Consciousness and cognition
(13) Mack et al. (2016) Consciousness and cognition
(14) Devue et al. (2009) Perception
(15) Memmert (2014) Cognitive Development
(16) Moore & Egeth (1997) JEP:HPP
(17) Cohen et al. (2020) Proc Natl Acad Sci
(18) Cohen et al. (2011) Psych Science

This is a critical point. The authors' key idea is that when you ask more than just a simple yes/no question, you find that other studies have overestimated the effects of inattentional blindness. But none of the studies listed above only asked simple yes/no questions. Thus, I believe the authors are mis-representing the field. Moreover, many of the studies that do much more than ask a simple yes/no question are cited by the authors themselves! Furthermore, as far as I can tell, the authors believe that if researchers do these extra steps and ask more follow-ups, then the results are valid. But since so many of these prior studies do those extra steps, I am not exactly sure what is being criticized.

To make sure this point is clear, I'd like to use a paper of mine as an example. In this study (Cohen et al., 2020, Proc Natl Acad Sci USA) we used gaze-contingent virtual reality to examine how much color people see in the world. On the critical trial, the part of the scene they fixated on was in color, but the periphery was entirely in black and white. As soon as the trial ended, we asked participants a series of questions to determine what they noticed. The list of questions included:

(1) "Did you notice anything strange or different about that last trial?"

(2) "If I were to tell you that we did something odd on the last trial, would you have a guess as to what we did?"

(3) "If I were to tell you we did something different in the second half of the last trial, would you have a guess as to what we did?"

(4) "Did you notice anything different about the colors in the last scene?"

(5) We then showed observers the previous trial again and drew their attention to the effect and confirmed that they did not notice that previously.

In a situation like this, when the observers are asked so many questions, do the authors believe that "the inattentionally blind can see after all?" I believe they would not say that and the reason they would not say that is because of the follow-up questions after the initial yes/no question. But since so many previous studies use similar follow-up questions, I do not think you can state that the field is broadly overestimating inattentional blindness. This is why it seems to me to be a bit of a straw-man: most people do not just use the yes/no method.

Main point 2: Let's imagine for a second that every study did just ask a yes/no question and then would stop. So, the criticism the authors are bringing up is valid (even though I believe it is not). I am not entirely sure that above chance performance on a forced choice task proves that the inattentionally blind can see after all. Could it just be a form of subliminal priming? Could there be a significant number of participants who basically would say something like, "No I did not see anything, and I feel like I am just guessing, but if you want me to say whether the thing was to the left or right, I will just 100% guess"? I know the literature on priming from things like change and inattentional blindness is a bit unclear, but this seems like maybe what is going on. In fact, maybe the authors are getting some of the best priming from inattentional blindness because of their large sample size, which previous studies do not use.

I'm curious how the authors would relate their studies to masked priming. In masked priming studies, observers say the did not see the target (like in this study) but still are above chance when forced to guess (like in this study). Do the researchers here think that that is evidence of "masked stimuli are truly seen" even if a participant openly says they are guessing?

Main point 3: My last question is about how the authors interpret a variety of inattentional blindness findings. Previous work has found that observers fail to notice a gorilla in a CT scan (Drew et al., 2013), a fight occurring right in front of them (Chabris et al., 2011), a plane on a runway that pilots crash into (Haines, 1991), and so forth. In a situation like this, do the authors believe that many participants are truly aware of these items but simply failed to answer a yes/no question correctly? For example, imagine the researchers made participants choose if the gorilla was in the left or right lung and some participants who initially said they did not notice the gorilla were still able to correctly say if it was in the left or right lung. Would the authors claim "that participant actually did see the gorilla in the lung"? I ask because it is difficult to understand what it means to be aware of something as salient as a gorilla in a CT scan, but say "no" you didn't notice it when asked a yes/no question. What does it mean to be aware of such important, ecologically relevant stimuli, but not act in response to them and openly say "no" you did not notice them?

Overall: I believe there are many aspects of this set of studies that are innovative and I hope the methods will be used more broadly in the literature. However, I believe the authors misrepresent the field and overstate what can be interpreted from their results. While I am sure there are cases where more nuanced questions might reveal inattentional blindness is somewhat overestimated, claims like "the inattentionally blind can see after all" or "Inattentionally blind subjects consciously perceive thest stimuli after all" seem to be incorrect (or at least not at all proven by this data).

# Author comment: Author response

**DOI:** 10.7554/eLife.100337.3.sa3

**Authors:** Makaela Nartker, Chaz Firestone, Howard Egeth, Ian Phillips

**Affiliations:** Johns Hopkins University Baltimore United States; Johns Hopkins University Baltimore United States; Johns Hopkins University Baltimore United States; Johns Hopkins University Baltimore United States

The following is the authors’ response to the current reviews.

**Responses to Reviewer #1:**

We thank the reviewer for these additional comments, and more generally for their extensive engagement with our work, which is greatly appreciated. Here, we respond to the three points in their latest review in turn.

The results of these experiments support a modest but important conclusion: If sub-optimal methods are used to collect retrospective reports, such as simple yes/no questions, inattentional blindness (IB) rates may be overestimated by up to ~8%.

It is true, of course, that we think the field has overstated the extent of IB, and we appreciate the reviewer characterizing our results as important along these lines. Nevertheless, we respectfully disagree with the framing and interpretation the reviewer attaches to them. As explained in our previous response, we think this interpretation — and the associated calculations of IB overestimation ‘rates’ — perpetuates a binary approach to perception and awareness which we regard as mistaken.

A graded approach to IB and visual awareness

Our sense is that many theorists interested in IB have conceived of perception and awareness as ‘all or nothing’: You either see a perfectly clear gorilla right in front of you, or you see nothing at all. This is implicit in the reviewer’s characterization of our results as simply indicating that fewer subjects fail to see the critical stimulus than previously assumed. To think that way is precisely to assume the orthodox binary position about perception, i.e., that any given subject can neatly be categorized into one of two boxes, *saw* or *didn’t see*.

Our perspective is different. We think there can be degraded forms of perception and awareness that fall neatly into neither of the categories “saw the stimulus perfectly clearly” or “saw nothing at all”. On this graded conception, the question is not: “What proportion of subjects saw the stimulus?” but: “What is the sensitivity of subjects to the stimulus?” This is why we prefer signal detection measures like *d′* over % noticing and % correct. This powerful framework has been successful in essentially every domain to which it has been applied, and we think perception and visual awareness are no exception. We understand that the reviewer may not think the same way about this foundational issue, but since part of our goal is to promote a graded approach to perception, we are keen to highlight our disagreement here and so resist the reviewer’s interpretation of our results (even to the extent that it is a positive one!).

Finally, we note that given this perspective, we are correspondingly inclined to reject many of the summary figures following below in Point (1) by the reviewer. These calculations (given in terms of % noticing and not noticing) make sense on the binary conception of awareness, but not on the SDT-based approach we favor. We say more about this below.

(1) In experiment 1, data from 374 subjects were included in the analysis. As shown in figure 2b, 267 subjects reported noticing the critical stimulus and 107 subjects reported not noticing it. This translates to a 29% IB rate if we were to only consider the "did you notice anything unusual Y/N" question. As reported in the results text (and figure 2c), when asked to report the location of the critical stimulus (left/right), 63.6% of the "non-noticer" group answered correctly. In other words, 68 subjects were correct about the location while 39 subjects were incorrect. Importantly, because the location judgment was a 2-alternative-forced-choice, the assumption was that if 50% (or at least not statistically different than 50%) of the subjects answered the location question correctly, everyone was purely guessing. Therefore, we can estimate that ~39 of the subjects who answered correctly were simply guessing (because 39 guessed incorrectly), leaving 29 subjects from the nonnoticer group who were correct on the 2AFC above and beyond the pure guess rate. If these 29 subjects are moved from the non-noticer to the noticer group, the corrected rate of IB for Experiment 1 is 20.86% instead of the original 28.61% rate that would have been obtained if only the Y/N question was used. In other words, relying only on the "Y/N did you notice anything" question led to an overestimate of IB rates by 7.75% in Experiment 1.

In the revised version of their manuscript, the authors provided the data that was missing from the original submission, which allows this same exercise to be carried out on the other 4 experiments.

(To briefly interject: All of these data were provided in our public archive since our original submission and remain available at https://osf.io/fcrhu. The difference now is only that they are included in the manuscript itself.)

Using the same logic as above, i.e., calculating the pure-guess rate on the 2AFC, moving the number of subjects above this pure-guess rate to the non-noticer group, and then re-calculating a "corrected IB rate", the other experiments demonstrate the following:

Experiment 2: IB rates were overestimated by 4.74% (original IB rate based only on Y/N question = 27.73%; corrected IB rate that includes the 2AFC = 22.99%)

Experiment 3: IB rates were overestimated by 3.58% (original IB rate = 30.85%; corrected IB rate = 27.27%)

Experiment 4: IB rates were overestimated by ~8.19% (original IB rate = 57.32%; corrected IB rate for color* = 39.71%, corrected IB rate for shape = 52.61%, corrected IB rate for location = 55.07%)

Experiment 5: IB rates were overestimated by ~1.44% (original IB rate = 28.99%; corrected IB rate for color = 27.56%, corrected IB rate for shape = 26.43%, corrected IB rate for location = 28.65%)

*note: the highest overestimate of IB rates was from Experiment 4, color condition, but the authors admitted that there was a problem with 2AFC color guessing bias in this version of the experiment which was a main motivation for running experiment 5 which corrected for this bias.

Taken as a whole, this data clearly demonstrates that even with a conservative approach to analyzing the combination of Y/N and 2AFC data, inattentional blindness was evident in a sizeable portion of the subject populations. An important (albeit modest) overestimate of IB rates was demonstrated by incorporating these improved methods.

We appreciate the work the reviewer has put into making these calculations. However, as noted above, such calculations implicitly reflect the binary approach to perception and awareness that we reject.

Consider how we’d think about the single subject case where the task is 2afc detection of a low contrast stimulus in noise. Suppose that this subject achieves 70% correct. One way of thinking about this is that the subject fully and clearly sees the stimulus on 40% of trials (achieving 100% correct on those) and guesses completely blindly on the other 60% (achieving 50% correct on those) for a total of 40% + 30% = 70% overall. However, this is essentially a ‘high threshold’ approach to the problem, in contrast to an SDT approach. On an SDT approach — an approach with tremendous evidential support — on every trial the subject receives samples from probabilistic distributions corresponding to each interval (one *noise* and one *signal + noise*) and determines which is higher according to the 2afc decision rule. Thus, across trials, they have access to differentially graded information about the stimulus. Moreover, on some trials they may have significant information from the stimulus (perhaps, well above their single interval detection criterion) but still decide incorrectly because of high noise from the other spatial interval. From this perspective, there is no nonarbitrary way of saying whether the subject saw/did not see on a given trial. Instead, we must characterize the subject’s overall sensitivity to the stimulus/its visibility to them in terms of a parameter such as *d′* (here, ~ 0.7).

We take the same attitude to the subjects in our experiments (and specifically to our ‘super subject’). Instead of calculating the proportion of subjects who saw or failed to see the stimulus (with some characterized as aware and some as unaware), we think the best way to characterize our results is that, across subjects (and so trials also), there was differential graded access to information from the stimulus, and this is best represented in terms of the group-level sensitivity parameter *d′*. This is why we frame our results as demonstrating that subjects traditionally considered inattentionally blind exhibit significant residual visual sensitivity to the critical stimulus.

(2) One of the strongest pieces of evidence presented in this paper was the single data point in Figure 3e showing that in Experiment 3, even the super subject group that rated their non-noticing as "highly confident" had a d' score significantly above zero. Asking for confidence ratings is certainly an improvement over simple Y/N questions about noticing, and if this result were to hold, it could provide a key challenge to IB. However, this result can most likely be explained by measurement error.

In their revised paper, the authors reported data that was missing from their original submission: the confidence ratings on the 2AFC judgments that followed the initial Y/N question. The most striking indication that this data is likely due to measurement error comes from the number of subjects who indicated that they were highly confident that they didn't notice anything on the critical trial, but then when asked to guess the location of the stimulus, indicated that they were highly confident that the stimulus was on the left (or right). There were 18 subjects (8.82% of the high-confidence non-noticer group) who responded this way. To most readers, this combination of responses (high confidence in correctly judging a stimulus feature that one is highly confident in having not seen at all) indicates that a portion of subjects misunderstood the confidence scales (or just didn't read the questions carefully or made mistakes in their responses, which is common for experiments conducted online).

In the authors' rebuttal to the first round of peer review, they wrote, "it is perfectly rationally coherent to be very confident that one didn't see anything but also very confident that if there was anything to be seen, it was on the left." I respectfully disagree that such a combination of responses is rationally coherent. The more parsimonious interpretation is that a measurement error occurred, and it's questionable whether we should trust any responses from these 18 subjects.

In their rebuttal, the authors go on to note that 14 of the 18 subjects who rated their 2AFC with high confidence were correct in their location judgment. If these 14 subjects were removed from analysis (which seems like a reasonable analysis choice, given their contradictory responses), d' for the high-confidence non-noticer group would most likely fall to chance levels. In other words, we would see a data pattern similar to that plotted in Figure 3e, but with the first data point on the left moving down to zero d'. This corrected Figure 3e would then provide a very nice evidence-based justification for including confidence ratings along with Y/N questions in future inattentional blindness studies.

We appreciate the reviewer’s highlighting of this particular piece of evidence as amongst our strongest. (At the same time, we must resist its characterization as a “single data point”: it derives from a large pre-registered experiment involving some 7,000 subjects total, with over 200 subjects in the relevant bin — both figures being far larger than a typical IB experiment.) We also appreciate their raising the issue of measurement error.

Specifically, the reviewer contends that our finding that even highly confident non-noticers exhibit significant sensitivity is “most likely … explained by measurement error” due to subjects mistakenly inverting our confidence scale in giving their response. In our original reply, we gave two reasons for thinking this quite unlikely; the reviewer has not addressed these in this revised review. First, we explicitly labeled our confidence scale (with 0 labeled as ‘Not at all confident’ and 3 as ‘Highly confident’) so that subjects would be very unlikely simply to invert the scale. This is especially so as it is very counterintuitive to treat “0” as reflecting *high* confidence. More importantly, however, we reasoned that any measurement error due to inverting or misconstruing the confidence scale should be symmetric. That is: If subjects are liable to invert the confidence scale, they should do so just as often when they answer “yes” as when they answer “no” – after all the very same scale is being used in both cases. This allows us to explore evidence of measurement error in relation to the large number of high-confidence “yes” subjects (N = 2677), thus providing a robust indicator as to whether subjects are generally liable to misconstrue the confidence scale. Looking at the number of such high confidence noticers who subsequently respond to the 2afc question with low confidence (a pattern which might, though need not, suggest measurement error), we found that the number was tiny. Only 28/2677 (1.05%) of high-confidence noticers subsequently gave the lowest level of confidence on the 2afc question, and only 63/2677 (2.35%) subjects gave *either* of the two lower levels of confidence. For these reasons, we consider any measurement error due to misunderstanding the confidence scale to be extremely minimal.

The reviewer is correct to note that 18/204 (9%) subjects reported both being highly confident that they didn't notice anything and highly confident in their 2afc judgment, although only 14/18 were correct in this judgment. Should we exclude these 14? Perhaps if we agree with the reviewer that such a pattern of responses is not “rationally coherent” and so must reflect a misconstrual of the scale. But such a pattern is in fact perfectly and straightforwardly intelligible. Specifically, in a 2afc task, two stimuli can individually fall well below a subject’s single interval detection criterion — leading to a high confidence judgment that nothing was presented in either interval. Quite consistent with this, the lefthand stimulus may produce a signal that is much higher than the right-hand stimulus — leading to a high confidence forced-choice judgment that, *if something was presented*, it was on the left. (By analogy, consider how a radiologist could look at a scan and say the following: “We’re 95% confident there’s no tumor. But even on the 5% chance that there is, our tests completely rule out that it’s a malignant one, so don’t worry.”)

(3) In most (if not all) IB experiments in the literature, a partial attention and/or full attention trial is administered after the critical trial. These control trials are very important for validating IB on the critical trial, as they must show that, when attended, the critical stimuli are very easy to see. If a subject cannot detect the critical stimulus on the control trial, one cannot conclude that they were inattentionally blind on the critical trial, e.g., perhaps the stimulus was just too difficult to see (e.g., too weak, too brief, too far in the periphery, too crowded by distractor stimuli, etc.), or perhaps they weren't paying enough attention overall or failed to follow instructions. In the aggregate data, rates of noticing the stimuli should increase substantially from the critical trial to the control trials. If noticing rates are equivalent on the critical and control trials, one cannot conclude that attention was manipulated in the first place.

In their rebuttal to the first round of peer review, the authors provided weak justification for not including such a control condition. They cite one paper that argues such control conditions are often used to exclude subjects from analysis (those who fail to notice the stimulus on the control trial are either removed from analysis or replaced with new subjects) and such exclusions/replacements can lead to underestimations of inattentional blindness rates. However, the inclusion of a partial or full attention condition as a control does not necessitate the extra step of excluding or replacing subjects. In the broadest sense, such a control condition simply validates the attention manipulation, i.e., one can easily compare the percent of subjects who answered "yes" or who got the 2AFC judgment correct during the critical trial versus the control trial. The subsequent choice about exclusion/replacement is separate, and researchers can always report the data with and without such exclusions/replacements to remain more neutral on this practice.

If anyone were to follow-up on this study, I highly recommend including a partial or full attention control condition, especially given the online nature of data collection. It's important to know the percent of online subjects who answer yes and who get the 2AFC question correct when the critical stimulus is attended, because that is the baseline (in this case, the "ceiling level" of performance) to which the IB rates on the critical trial can be compared.

We agree with the reviewer that future studies could benefit from including a partial or full attention condition. They are surely right that we might learn something additional from such conditions.

Where we differ from the reviewer is in thinking of these conditions as “controls” appropriate to our research question. This is why we offered the justification we did in our earlier response. *When these conditions are used as controls*, they are used to exclude subjects in ways that serve to inflate the biases we are concerned with in our work. For our question, the absence of these conditions does not impact the significance of the findings, since such conditions are designed to answer a question which is not the one at the heart of our paper. Our key claim is that subjects who deny noticing an unexpected stimulus in a standard inattentional blindness paradigm nonetheless exhibit significant residual sensitivity (as well as a conservative bias in their response to the noticing question); the presence or absence of partial- or full-attention conditions is orthogonal to that question.

Moreover, we note that our tasks were precisely chosen to be classic tasks widely used in the literature to manipulate attention. Thus, by common consensus in the field, they are effective means to soak up attention, and have in effect been tested in partial- and full-attention control settings in a huge number of studies. Second, we think it very doubtful that subjects in a full-attention trial would not overwhelmingly have detected our critical stimuli. The reviewer worries that they might have been “too weak, too brief, too far in the periphery, too crowded by distractor stimuli, etc.” But consider E5 where the stimulus was a highly salient orange or green shape, present on the screen for 5 seconds. The reviewer also suggests that subjects in the full-attention control might not have detected the stimulus because they “weren't paying enough attention overall”. But evidently if they weren’t paying attention even in the full-attention trial this would be reason for thinking that there was inattentional blindness even in this condition (a point made by White et al. 2018) and certainly not a reason for thinking there was not an attentional effect in the critical trial. Lastly, the reviewer suggests that a full-attention condition would have helped ensure that subjects were following instructions. But we ensured this already by (as per our pre-registration) excluding subjects who performed poorly in the relevant primary tasks.

Thus, both in principle and in practice, we do not see the absence of such conditions as impacting the interpretation of our findings, even as we agree that future work posing a different research question could certainly learn something from including such conditions.

**Responses to Reviewer #2:**

We note that this report is unchanged from an earlier round of review, and not a response to our significantly revised manuscript. We believe our latest version fully addresses all the issues which the reviewer originally raised. The interested reader can see our original response below. We again thank the reviewer for their previous report which was extremely helpful.

—-

The following is the authors’ response to the original reviews.

**eLife Assessment**

This study presents valuable findings to the field interested in inattentional blindness (IB), reporting that participants indicating no awareness of unexpected stimuli through yes/no questions, still show above-chance sensitivity to specific properties of these stimuli through follow-up forced-choice questions (e.g., its color). The results suggest that this is because participants are conservative and biased to report not noticing in IB. The authors conclude that these results provide evidence for residual perceptual awareness of inattentionally blind stimuli and that therefore these findings cast doubt on the claim that awareness requires attention. Although the samples are large and the analysis protocol novel, the evidence supporting this interpretation is still incomplete, because effect sizes are rather small, the experimental design could be improved and alternative explanations have not been ruled out.

We are encouraged to hear that *eLife* found our work “valuable”. We also understand, having closely looked at the reviews, why the assessment also includes an evaluation of “incomplete”. We gave considerable attention to this latter aspect of the assessment in our revision. In addition to providing additional data and analyses that we believe strengthen our case, we also include a much more substantial review and critique of existing methods in the IB literature to make clear exactly the gap our work fills and the advance it makes. (Indeed, if it is appropriate to say this here, we believe one key aspect of our work that is missing from the assessment is our inclusion of ‘absent’ trials, which is what allows us to make the crucial claims about conservative reporting of awareness in IB for the first time.) Moreover, we refocus our discussion on only our most central claims, and weaken several of our secondary claims so that the data we’ve collected are better aligned with the conclusions we draw, to ensure that the case we now make is in fact complete. Specifically, our two core claims are (1) that there is residual sensitivity to visual features for subjects who would ordinarily be classified as inattentionally blind (whether this sensitivity is conscious or not), and (2) that there is a tendency to respond conservatively on yes/no questions in the context of IB. We believe we have very compelling support for these two core claims, as we explain in detail below and also through revisions to our manuscript.

Given the combination of strengthened and clarified case, as well as the weakening of any conclusions that may not have been fully supported, we believe and hope that these efforts make our contribution “solid”, “convincing”, or even “compelling” (especially because the “compelling” assessment characterizes contributions that are “more rigorous than the current state-of-the-art”, which we believe to be the case given the issues that have plagued this literature and that we make progress on).

**Reviewer #1 (Public review):**

Summary:

In the abstract and throughout the paper, the authors boldly claim that their evidence, from the largest set of data ever collected on inattentional blindness, supports the views that "inattentionally blind participants can successfully report the location, color, and shape of stimuli they deny noticing", "subjects retain awareness of stimuli they fail to report", and "these data...cast doubt on claims that awareness requires attention." If their results were to support these claims, this study would overturn 25+ years of research on inattentional blindness, resolve the rich vs. sparse debate in consciousness research, and critically challenge the current majority view in cognitive science that attention is necessary for awareness.

Unfortunately, these extraordinary claims are not supported by extraordinary (or even moderately convincing) evidence. At best, the results support the more modest conclusion: If sub-optimal methods are used to collect retrospective reports, inattentional blindness rates will be overestimated by up to ~8% (details provided below in comment #1). This evidence-based conclusion means that the phenomenon of inattentional blindness is alive and well as it is even robust to experiments that were specifically aimed at falsifying it. Thankfully, improved methods already exist for correcting the ~8% overestimation of IB rates that this study successfully identified.

We appreciate here the reviewer’s recognition of the importance of work on inattentional blindness, and the centrality of inattentional blindness to a range of major questions. We also recognize their concerns with what they see as a gap between our data and the claims made on their basis. We address this in detail below (as well as, of course, in our revised manuscript). However, from the outset we are keen to clarify that our central claim is only the first one the reviewer mentions — and the one which appears in our title — namely that, as a group, participants can successfully report the location, color, and shape of stimuli they deny noticing, and thus that there is “Sensitivity to visual features in inattentional blindness”. This is the claim that we believe is strongly supported by our data, and all the more so after revising the manuscript in light of the helpful comments we’ve received.

By contrast, the other claims the reviewer mentions, concerning awareness (as opposed to residual sensitivity–which might be conscious or unconscious) were intended as both secondary and tentative. We agree with the referee that these are not as strongly supported by our data (and indeed we say so in our manuscript), whereas we do think our data strongly support the more modest — and, to us central — claim that, as a group, inattentionally blind participants can successfully report the location, color, and shape of stimuli they deny noticing.

We also feel compelled to resist somewhat the reviewer’s summary of our claims. For example, the reviewer attributes to us the claim that “subjects retain awareness of stimuli they fail to report”; but while that phrase does appear in our abstract, what we in fact say is that our data are “**consistent with** an alternative hypothesis about IB, namely that subjects retain awareness of stimuli they fail to report”. We do in fact believe that our data are consistent with that hypothesis, whereas earlier investigations seemed not to be. We mention this only because we had used that careful phrasing precisely for this sort of reason, so that we *wouldn’t* be read as saying that our results unequivocally support that alternative.

Still, looking back, we see how we may have given more emphasis than we intended to some of these more secondary claims. So, we’ve now gone through and revised our manuscript throughout to emphasize that our main claim is about residual sensitivity, and to make clear that our claims about awareness are secondary and tentative. Indeed, we now say precisely this, that although we favor an interpretation of “our results in terms of residual conscious vision in IB … this claim is tentative and secondary to our primary finding”. We also weaken the statements in the abstract that the reviewer mentions, to better reflect our key claims.

Finally, we note one further point: Dialectically, inattentional blindness has been used to argue (e.g.) that attention is required for awareness. We think that our data concerning residual sensitivity at least push back on the *use* of IB to make this claim, even if (as we agree) they do not provide decisive evidence that awareness survives inattention. In other words, we think our data call that claim into question, such that it’s now genuinely unclear whether awareness does or does not survive inattention. We have adjusted our claims on this point accordingly as well.

Comments:

(1) In experiment 1, data from 374 subjects were included in the analysis. As shown in figure 2b, 267 subjects reported noticing the critical stimulus and 107 subjects reported not noticing it. This translates to a 29% IB rate, if we were to only consider the "did you notice anything unusual Y/N" question. As reported in the results text (and figure 2c), when asked to report the location of the critical stimulus (left/right), 63.6% of the "non-noticer" group answered correctly. In other words, 68 subjects were correct about the location while 39 subjects were incorrect. Importantly, because the location judgment was a 2-alternative-forced-choice, the assumption was that if 50% (or at least not statistically different than 50%) of the subjects answered the location question correctly, everyone was purely guessing. Therefore, we can estimate that ~39 of the subjects who answered correctly were simply guessing (because 39 guessed incorrectly), leaving 29 subjects from the nonnoticer group who may have indeed actually seen the location of the stimulus. If these 29 subjects are moved to the noticer group, the corrected rate of IB for experiment 1 is 21% instead of 29%. In other words, relying only on the "Y/N did you notice anything" question leads to an overestimate of IB rates by 8%. This modest level of inaccuracy in estimating IB rates is insufficient for concluding that "subjects retain awareness of stimuli they fail to report", i.e. that inattentional blindness does not exist.

In addition, this 8% inaccuracy in IB rates only considers one side of the story. Given the data reported for experiment 1, one can also calculate the number of subjects who answered "yes, I did notice something unusual" but then reported the incorrect location of the critical stimulus. This turned out to be 8 subjects (or 3% of the "noticer" group). Some would argue that it's reasonable to consider these subjects as inattentionally blind, since they couldn't even report where the critical stimulus they apparently noticed was located. If we move these 8 subjects to the non-noticer group, the 8% overestimation of IB rates is reduced to 6%.

The same exercise can and should be carried out on the other 4 experiments, however, the authors do not report the subject numbers for any of the other experiments, i.e., how many subjects answered Y/N to the noticing question and how many in each group correctly answered the stimulus feature question. From the limited data reported (only total subject numbers and d' values), the effect sizes in experiments 2-5 were all smaller than in experiment 1 (d' for the non-noticer group was lower in all of these follow-up experiments), so it can be safely assumed that the ~6-8% overestimation of IB rates was smaller in these other four experiments. In a revision, the authors should consider reporting these subject numbers for all 5 experiments.

We now report, as requested, all these subject numbers in our supplementary data (see Supplementary Tables 1 and 2 in our Supplementary Materials).

However, we wish to address the larger question the reviewer has raised: Do our data only support a relatively modest reduction in IB rates? Even if they did, we *still* believe that this would be a consequential result, suggesting a significant overestimation of IB rates in classic paradigms. However, part of our purpose in writing this paper is to push back against a certain binary way of thinking about seeing/awareness. Our sense is that the field has conceived of awareness as “all or nothing”: You either see a perfectly clear gorilla right in front of you, or you see nothing at all. Our perspective is different: We think there can be degraded forms of awareness that fall into neither of those categories. For that reason, we are disinclined to see our results in the way that the reviewer suggests, namely as simply indicating that fewer subjects fail to see the stimulus than previously assumed. To think that way is, in our view, to assume the orthodox binary position about awareness. If, instead, one conceives of awareness as we do (and as we believe the framework of signal detection theory should compel us to), then it isn’t quite right to think of the proportion of subjects who were aware, but rather (e.g.) the sensitivity of subjects to the relevant stimulus. This is why we prefer measures like d′ over % noticing and % correct. We understand that the reviewer may not think the same way about this issue as we do, but part of our goal is to promote that way of thinking in general, and so some of our comments below reflect that perspective and approach.

For example, consider how we’d think about the single subject case where the task is 2afc detection of a low contrast stimulus in noise. Suppose that this subject achieves 70% correct. One way of thinking about that is that the subject sees the stimulus on 40% of trials (achieving 100% correct on those) and guesses blindly on the other 60% (achieving 50% correct on those) for a total of 40% + 30% = 70% overall. However, this is essentially a “high threshold” approach to the problem, in contrast to an SDT approach. On an SDT approach (an approach with tremendous evidential support), on every trial the subject receives samples from probabilistic distributions corresponding to each interval (one noise and one signal + noise) and determines which is higher according to the 2afc decision rule. Thus, across trials they have access to differentially graded information about the stimulus. Moreover, on some trials they may have significant information from the stimulus (perhaps, well above their single interval detection criterion) but still decide incorrectly because of high noise from the other spatial interval. From this perspective, there is no non-arbitrary way of saying whether the subject saw/did not see on a given trial. Instead, we must characterize the subject’s overall sensitivity to the stimulus/its visibility to them in terms of a parameter such as *d′* (here, ~ 0.7).

We take the same attitude to our super subject. Instead of saying that some subjects saw/failed to see the stimuli, instead we suggest that the best way to characterize our results is that across subjects (and so trials also) there was differential graded access to information from the stimulus best represented in terms of the group-level sensitivity parameter d′.

We acknowledge that (despite ourselves) we occasionally fell into an all-too-natural binary/high threshold way of thinking, as when we suggested that our data show that “inattentionally blind subjects consciously perceive these stimuli after all” and “the inattentionally blind can see after all." (p.17) We have removed such problematic phrasing as well as other problematic phrasing as noted below.

(2) Because classic IB paradigms involve only one critical trial per subject, the authors used a "super subject" approach to estimate sensitivity (d') and response criterion (c) according to signal detection theory (SDT). Some readers may have issues with this super subject approach, but my main concern is with the lack of precision used by the authors when interpreting the results from this super subject analysis.

Only the super subject had above-chance sensitivity (and it was quite modest, with d' values between 0.07 and 0.51), but the authors over-interpret these results as applying to every subject. The methods and analyses cannot determine if any individual subject could report the features above-chance. Therefore, the following list of quotes should be revised for accuracy or removed from the paper as they are misleading and are not supported by the super subject analysis: "Altogether this approach reveals that subjects can report above-chance the features of stimuli (color, shape, and location) that they had claimed not to notice under traditional yes/no questioning" (p.6)

"In other words, nearly two-thirds of subjects who had just claimed not to have noticed any additional stimulus were then able to correctly report its location." (p.6)

"Even subjects who answer "no" under traditional questioning can still correctly report various features of the stimulus they just reported not having noticed, suggesting that they were at least partially aware of it after all." (p.8)

"Why, if subjects could succeed at our forced-response questions, did they claim not to have noticed anything?" (p.8)

"we found that observers could successfully report a variety of features of unattended stimuli, even when they claimed not to have noticed these stimuli." (p.14)

"our results point to an alternative (and perhaps more straightforward) explanation: that inattentionally blind subjects consciously perceive these stimuli after all... they show sensitivity to IB stimuli because they can see them." (p.16)

"In other words, the inattentionally blind can see after all." (p.17)

We thank the reviewer for pointing out how these quotations may be misleading as regards our central claim. We intended them all to be read generically as concerning the group, and not universally as claiming that all subjects could report above-chance/see the stimuli etc. We agree entirely that the latter universal claim would not be supported by our data. In contrast, we do contend that our super-subject analysis shows that, *as a group*, subjects traditionally considered intentionally blind exhibit residual sensitivity to features of stimuli (color, shape, and location) that they had all claimed not to notice, and likewise that *as a group* they could succeed at our forced-choice questions.

To ensure this claim is clear throughout the paper, and that we are not interpreted as making an unsupported universal claim we have revised the language in all of the quotations above, as follows, as well as in numerous other places in the paper.

“Altogether this approach reveals that subjects can report above-chance the features of stimuli (color, shape, and location) that they had claimed not to notice under traditional yes/no questioning” (p.6) => “Altogether this approach reveals that as a group subjects can report above-chance the features of stimuli (color, shape, and location) that they had all claimed not to notice under traditional yes/no questioning” (p.6)

“Even subjects who answer “no” under traditional questioning can still correctly report various features of the stimulus they just reported not having noticed, suggesting that they were at least partially aware of it after all.” (p.8) => “... even subjects who answer “no” under traditional questioning can, as a group, still correctly report various features of the stimuli they just reported not having noticed, indicating significant group-level sensitivity to visual features. Moreover, these results are even consistent with an alternative hypothesis about IB, that as a group, subjects who would traditionally be classified as inattentionally blind are in fact at least partially aware of the stimuli they deny noticing.” (p.8)

“Why, if subjects could succeed at our forced-response questions, did they claim not to have noticed anything?” (p.8) => “Why, if subjects could succeed at our forcedresponse questions as a group, did they all individually claim not to have noticed anything?” (p.8)

“we found that observers could successfully report a variety of features of unattended stimuli, even when they claimed not to have noticed these stimuli.” (p.14) => “we found that groups of observers could successfully report a variety of features of unattended stimuli, even when they all individually claimed not to have noticed those stimuli.” (p.14)

“our results point to an alternative (and perhaps more straightforward) explanation: that inattentionally blind subjects consciously perceive these stimuli after all... they show sensitivity to IB stimuli because they can see them.” (p.16) => “our results just as easily raise an alternative (and perhaps more straightforward) explanation: that inattentionally blind subjects may retain a degree of awareness of these stimuli after all.” (p.16) Here deleting: “they show sensitivity to IB stimuli because they can see them.”

“In other words, the inattentionally blind can see after all.” (p.17) => “In other words, as a group, the inattentionally blind enjoy at least some degraded or partial sensitivity to the location, color and shape of stimuli which they report not noticing.” (p.17)

In one case, we felt the sentence was correct as it stood, since it simply reported a fact about our data:

“In other words, nearly two-thirds of subjects who had just claimed not to have noticed any additional stimulus were then able to correctly report its location.” (p.6)

After all, if subjects were entirely blind and simply guessed, it would be true to say that 50% of subjects would be able to correctly report the stimulus location (by guessing).

In addition to these and numerous other changes, we also added the following explicit statement early in the paper to head-off any confusion on this point: “Note that all analyses reported here relate to this super subject as opposed to individual subjects”.

(3) In addition to the d' values for the super subject being slightly above zero, the authors attempted an analysis of response bias to further question the existence of IB. By including in some of their experiments critical trials in which no critical stimulus was presented, but asking subjects the standard Y/N IB question anyway, the authors obtained false alarm and correct rejection rates. When these FA/CR rates are taken into account along with hit/miss rates when critical stimuli were presented, the authors could calculate c (response criterion) for the super subject. Here, the authors report that response criteria are biased towards saying "no, I didn't notice anything". However, the validity of applying SDT to classic Y/N IB questioning is questionable.

For example, with the subject numbers provided in Box 1 (the 2x2 table of hits/misses/FA/CR), one can ask, 'how many subjects would have needed to answer "yes, I noticed something unusual" when nothing was presented on the screen in order to obtain a non-biased criterion estimate, i.e., c = 0?' The answer turns out to be 800 subjects (out of the 2761 total subjects in the stimulus-absent condition), or 29% of subjects in this condition.

In the context of these IB paradigms, it is difficult to imagine 29% of subjects claiming to have seen something unusual when nothing was presented. Here, it seems that we may have reached the limits of extending SDT to IB paradigms, which are very different than what SDT was designed for. For example, in classic psychophysical paradigms, the subject is asked to report Y/N as to whether they think a threshold-level stimulus was presented on the screen, i.e., to detect a faint signal in the noise. Subjects complete many trials and know in advance that there will often be stimuli presented and the stimuli will be very difficult to see. In those cases, it seems more reasonable to incorrectly answer "yes" 29% of the time, as you are trying to detect something very subtle that is out there in the world of noise. In IB paradigms, the stimuli are intentionally designed to be highly salient (and unusual), such that with a tiny bit of attention they can be easily seen. When no stimulus is presented and subjects are asked about their own noticing (especially of something unusual), it seems highly unlikely that 29% of them would answer "yes", which is the rate of FAs that would be needed to support the null hypothesis here, i.e., of a non-biased criterion. For these reasons, the analysis of response bias in the current context is questionable and the results claiming to demonstrate a biased criterion do not provide convincing evidence against IB.

We are grateful to the reviewer for highlighting this aspect of our data. We agree with several of these points. For example, it is indeed striking that — given the corresponding hit rate — a false alarm rate of 29% would be needed to obtain an unbiased criterion. At the same time, we would respectfully push back on other points above. In our first experiment that uses the super-subject analysis, for example, *d′* is 0.51 and highly significant; to describe that figure, as the reviewer does, as “*slightly above zero*” seemed not quite right to us (and all the more so given that these experiments involve very large samples and preregistered analysis plans).

We also respectfully disagree that our data call into question the validity of applying SDT to classic yes/no IB questioning. The mathematical foundations of SDT are rock solid, and have been applied far more broadly than we have applied them here. In fact, in a way we would suggest that exactly the opposite attitude is appropriate: rather than thinking that IB challenges an immensely well-supported, rigorously tested and broadly applicable mathematical model of perception, we think that the conflict between our SDT-based model of IB and the standard interpretation constitutes strong reason to disfavor the standard interpretation. Several points are worth making here.

First, it is already surprising that 11.03% of our subjects in E2 (46/417) and 7.24% of our subjects in E5 (200/2761) E5 reported noticing a stimulus when no stimulus was present. But while this may have seemed unlikely in advance of inquiry, this is in fact what the data show and forms the basis of our criterion calculations. Thus, our criterion calculations already factor in a surprising but empirically verified high false alarm rate of subjects answering “yes” when no stimulus was presented and were asked about their noticing. (We also note that the only paper we know of to report a false alarm rate in an IB paradigm, though not one used to calculate a response criterion, found a very consistent false alarm rate of 10.4%. See Devue et al. 2009.)

Second, while the reviewer is of course correct that a common psychophysical paradigm involves detection of a “threshold-level”/faint stimulus in noise, it is widely recognized that SDT has an extremely broad application, being applicable to any situation in which two kinds of event are to be discriminated (Pastore & Scheirer 1975) and being “almost universally accepted as a theoretical account of decision making in research on perceptual detection and recognition and in numerous extensions to applied domains” quite generally (Estes 2002, see also: Wixted 2020). Indeed, cases abound in which SDT has been successfully applied to situations which do not involve near threshold stimuli in noise. To pick two examples at random, SDT has been used in studying acceptability judgments in linguistics (Huang and Ferreira 2020) and the assessment of physical aggression in childstudent interactions (Lerman et al. 2010; for more general discussion of practical applications, see Swets et al. 2000). Given that the framework of SDT is so widely applied and well supported, and that we see no special reason to make an exception, we believe it can be relied on in the present context.

Finally, we note that inattentional blindness can in many ways be considered analogous to “near threshold” detection since inattention is precisely thought to degrade or even abolish awareness of stimuli, meaning that our stimuli can be construed as near threshold in the relevant sense. Indeed, our relatively modest d′ values suggest that under inattention stimuli are indeed hard to detect. Thus, even were SDT more limited in its application, we think it still would be appropriate to apply to the case of IB.

(4) One of the strongest pieces of evidence presented in the entire paper is the single data point in Figure 3e showing that in Experiment 3, even the super subject group that rated their non-noticing as "highly confident" had a d' score significantly above zero. Asking for confidence ratings is certainly an improvement over simple Y/N questions about noticing, and if this result were to hold, it could provide a key challenge to IB. However, this result hinges on a single data point, it was not replicated in any of the other 4 experiments, and it can be explained by methodological limitations. I strongly encourage the authors (and other readers) to follow up on this result, in an in-person experiment, with improved questioning procedures.

We agree that our finding that even the super-subject group that rated their non-noticing as “highly confident” had a *d'* score significantly above zero is an especially strong piece of evidence, and we thank the reviewer for highlighting that here. At the same time, we note that while the finding is represented by a single marker in Figure 3e, it seemed not quite right to call this a “single data point”, as the reviewer does, given that it derives from a large pre-registered experiment involving some 7,000 subjects total, with over 200 subjects in the relevant bin — both figures being far larger than a typical IB experiment. It would of course be tremendous to follow up on this result – and we certainly hope our work inspires various follow-up studies. That said, we note that recruiting the necessary numbers of in person subjects would be an absolutely enormous, career-level undertaking – it would involve bringing more than the entire undergraduate population at our own institution, Johns Hopkins, into our laboratory! While those results would obviously be extremely valuable, we wouldn’t want to read the reviewer’s comments as implying that only an experiment of that magnitude — requiring thousands upon thousands of in-person subjects — could make progress on these issues. Indeed, because every subject can only contribute one critical trial in IB, it has long been recognized as an extremely challenging paradigm to study in a sufficiently well-powered and psychophysically rigorous way. We believe that our large preregistered online approach represents a major leap forward here, even if it involves certain trade-offs.

In the current Experiment 3, the authors asked the standard Y/N IB question, and then asked how confident subjects were in their answer. Asking back-to-back questions, the second one with a scale that pertains to the first one (including a tricky inversion, e.g., "yes, I am confident in my answer of no"), may be asking too much of some subjects, especially subjects paying half-attention in online experiments. This procedure is likely to introduce a sizeable degree of measurement error.

An easy fix in a follow-up study would be to ask subjects to rate their confidence in having noticed something with a single question using an unambiguous scale:

On the last trial, did you notice anything besides the cross?

(1): I am highly confident I didn't notice anything else

(2): I am confident I didn't notice anything else

(3): I am somewhat confident I didn't notice anything else

(4): I am unsure whether I noticed anything else

(5): I am somewhat confident I noticed something else

(6): I am confident I noticed something else

(7): I am highly confident I noticed something else

If we were to re-run this same experiment, in the lab where we can better control the stimuli and the questioning procedure, we would most likely find a d' of zero for subjects who were confident or highly confident (1-2 on the improved scale above) that they didn't notice anything. From there on, the d' values would gradually increase, tracking along with the confidence scale (from 3-7 on the scale). In other words, we would likely find a data pattern similar to that plotted in Figure 3e, but with the first data point on the left moving down to zero d'. In the current online study with the successive (and potentially confusing) retrospective questioning, a handful of subjects could have easily misinterpreted the confidence scale (e.g., inverting the scale) which would lead to a mixture of genuine high-confidence ratings and mistaken ratings, which would result in a super subject d' that falls between zero and the other extreme of the scale (which is exactly what the data in Fig 3e shows).

One way to check on this potential measurement error using the existing dataset would be to conduct additional analyses that incorporate the confidence ratings from the 2AFC location judgment task. For example, were there any subjects who reported being confident or highly confident that they didn't see anything, but then reported being confident or highly confident in judging the location of the thing they didn't see? If so, how many? In other words, how internally (in)consistent were subjects' confidence ratings across the IB and location questions? Such an analysis could help screen-out subjects who made a mistake on the first question and corrected themselves on the second, as well as subjects who weren't reading the questions carefully enough.

As far as I could tell, the confidence rating data from the 2AFC location task were not reported anywhere in the main paper or supplement.

We are grateful to the reviewer for raising this issue and for requesting that we report the confidence rating data from our 2afc location task in Experiment 3. We now report all this data in our Supplementary Materials (see Supplementary Table 3).

We of course agree with the reviewer’s concern about measurement error, which is a concern in all experiments. What, then, of the particular concern that some subjects might have misunderstood our confidence question? It is surely impossible in principle to rule out this possibility; however, several factors bear on the plausibility of this interpretation. First, we explicitly labeled our confidence scale (with 0 labeled as ‘Not at all confident’ and 3 as ‘Highly confident’) so that subjects would be very unlikely simply to invert the scale. This is especially so as it is very counterintuitive to treat “0” as reflecting *high* confidence. However, we accept that it is a possibility that certain subjects might nonetheless have been confused in some other way.

So, we also took a second approach. We examined the confidence ratings on the 2afc question of subjects who reported being highly confident that they didn't notice anything.

Reassuringly, the large majority of these high confidence “no” subjects (~80%) reported low confidence of 0 or 1 on the 2afc question, and the majority (51%) reported the lowest confidence of 0. Only 18/204 (9%) subjects reported high confidence on both questions.

Still, the numbers of subjects here are small and so may not be reliable. This led us to take a third approach. We reasoned that any measurement error due to inverting or misconstruing the confidence scale should be symmetric. That is: If subjects are liable to invert the confidence scale, they should do so just as often when they answer “yes” as when they answer “no” – after all the very same scale is being used in both cases. This allows us to explore evidence of measurement error in relation to the much larger number of highconfidence “yes” subjects (N = 2677), thus providing a much more robust indicator as to whether subjects are generally liable to misconstrue the confidence scale. Looking at the number of such high confidence noticers who subsequently respond to the 2afc question with low-confidence, we found that the number was tiny. Only 28/2677 (1.05%) of highconfidence noticers subsequently gave the lowest level of confidence on the 2afc question, and only 63/2677 (2.35%) subjects gave *either* of the two lower levels of confidence. In this light, we consider any measurement error due to misunderstanding the confidence scale to be extremely minimal.

What should we make of the 18 subjects who were highly confident non-noticers but then only low-confidence on the 2afc question? Importantly, we do not think that these 18 subjects necessarily made a mistake on the first question and so should be excluded. There is no a priori reason why one’s confidence criterion in a yes/no question should carry over to a 2afc question. After all, it is perfectly rationally coherent to be very confident that one didn’t see anything but also very confident that *if* there was anything to be seen, it was on the left. Moreover, these 18 subjects were *not* all correct on the 2afc question despite their high confidence (4/18 or 22% getting the wrong answer).

Nonetheless, and again reassuringly, we found that the above-chance patterns in our data remained the same even excluding these 18 subjects. We did observe a slight reduction in percent correct and *d′* but this is absolutely what one should expect since excluding the most confident performers in any task will almost inevitably reduce performance.

In this light, we consider it unlikely that measurement error fully explains the residual sensitivity found even amongst highly confident non-noticers. That said, we appreciate this concern. We now raise the issue and the analysis of high confidence noticers which addresses it in our revised manuscript. We also thank the reviewer for pressing us to think harder about this issue, which led directly to these new analyses that we believed have strengthened the paper.

(5) In most (if not all) IB experiments in the literature, a partial attention and/or full attention trial (or set of trials) is administered after the critical trial. These control trials are very important for validating IB on the critical trial, as they must show that, when attended, the critical stimuli are very easy to see. If a subject cannot detect the critical stimulus on the control trial, one cannot conclude that they were inattentionally blind on the critical trial, e.g., perhaps the stimulus was just too difficult to see (e.g., too weak, too brief, too far in the periphery, too crowded by distractor stimuli, etc.), or perhaps they weren't paying enough attention overall or failed to follow instructions. In the aggregate data, rates of noticing the stimuli should increase substantially from the critical trial to the control trials. If noticing rates are equivalent on the critical and control trials one cannot conclude that attention was manipulated.

It is puzzling why the authors decided not to include any control trials with partial or full attention in their five experiments, especially given their online data collection procedures where stimulus size, intensity, eccentricity, etc. were uncontrolled and variable across subjects. Including such trials could have actually helped them achieve their goal of challenging the IB hypothesis, e.g., excluding subjects who failed to see the stimulus on the control trials might have reduced the inattentional blindness rates further. This design decision should at least be acknowledged and justified (or noted as a limitation) in a revision of this paper.

We acknowledge that other studies in the literature include divided and full attention trials, and that they could have been included in our work as well. However, we deliberately decided not to include such control trials for an important reason. As the referee comments, the main role of such trials in previous work has been to exclude from analysis subjects who failed to report the unexpected stimulus on the divided and/or full attention control trials.

(For example, as Most et al. 2001 write: “Because observers should have seen the object in the full-attention trial (Mack & Rock, 1998), we used this trial as a control … Accordingly, 3 observers who failed to see the cross on this trial were replaced, and their data were excluded from the analyses.") As the reviewer points out, excluding such subjects would very likely have ‘helped' us. However, the practice is controversial. Indeed, in a review of 128 experiments, White et al. 2018 argue that the practice has “problematic consequences” and “may lead researchers to understate the pervasiveness of inattentional blindness". Since we wanted to offer as simple and demanding a test of residual sensitivity in IB as possible, we thus decided not to use any such exclusions, and for that reason decided not to include divided/full attention trials.

As recommended, we discuss this decision not to include divided/full attention trials and our logic for not doing so in the manuscript. As we explain, not having those conditions makes it more impressive, not less impressive, that we observed the results we in fact did — it makes our results more interpretable, not less interpretable, and so absence of such conditions from our manuscript should not (in our view) be considered any kind of weakness.

(6) In the discussion section, the authors devote a short paragraph to considering an alternative explanation of their non-zero d' results in their super subject analyses: perhaps the critical stimuli were processed unconsciously and left a trace such that when later forced to guess a feature of the stimuli, subjects were able to draw upon this unconscious trace to guide their 2AFC decision. In the subsequent paragraph, the authors relate these results to above-chance forced-choice guessing in blindsight subjects, but reject the analogy based on claims of parsimony.

First, the authors dismiss the comparison of IB and blindsight too quickly. In particular, the results from experiment 3, in which some subjects adamantly (confidently) deny seeing the critical stimulus but guess a feature at above-chance levels (at least at the super subject level and assuming the online subjects interpreted and used the confidence scale correctly), seem highly analogous to blindsight. Importantly, the analogy is strengthened if the subjects who were confident in not seeing anything also reported not being confident in their forced-choice judgments, but as mentioned above this data was not reported.

Second, the authors fail to mention an even more straightforward explanation of these results, which is that ~8% of subjects misinterpreted the "unusual" part of the standard IB question used in experiments 1-3. After all, colored lines and shapes are pretty "usual" for psychology experiments and were present in the distractor stimuli everyone attended to. It seems quite reasonable that some subjects answered this first question, "no, I didn't see anything unusual", but then when told that there was a critical stimulus and asked to judge one of its features, adjusted their response by reconsidering, "oh, ok, if that's the unusual thing you were asking about, of course I saw that extra line flash on the left of the screen". This seems like a more parsimonious alternative compared to either of the two interpretations considered by the authors: (1) IB does not exist, (2) super-subject d' is driven by unconscious processing. Why not also consider: (3) a small percentage of subjects misinterpreted the Y/N question about noticing something unusual. In experiments 4-5, they dropped the term "unusual" but do not analyze whether this made a difference nor do they report enough of the data (subject numbers for the Y/N question and 2AFC) for readers to determine if this helped reduce the ~8% overestimate of IB rates.

Our primary ambition in the paper was to establish, as our title suggests, residual sensitivity in IB. The ambition is quite neutral as to whether the sensitivity reflects conscious or unconscious processing (i.e. is akin to blindsight as traditionally conceived). We were evidently not clear about this, however, leading to two referees coming away with an impression of our claims that is different than we intended. We have revised our manuscript throughout to address this. But we also want to emphasize here that we take our data primarily to support the more modest claim that there is residual sensitivity (conscious or unconscious) in the group of subjects who are traditionally classified as inattentionally blind. We believe that this claim has solid support in our data.

We do in the discussion section offer one reason for believing that there is residual awareness in the group of subjects who are traditionally classified as inattentionally blind. However, we acknowledge that this is controversial and now emphasize in the manuscript that this claim “is tentative and secondary to our primary finding”. We also emphasize that part of our point is dialectical: Inattentional blindness has been used to argue (e.g.) that attention is required for awareness. We think that our data concerning residual sensitivity at least push back on the *use* of IB to make this claim, even if they do not provide decisive evidence (as we agree) that awareness survives inattention. (Cf. here, Hirshhorn et al. 2024 who take up a common suggestion in the field that awareness is best assessed by using both subjective and objective measures, with claims about lack of awareness ideally being supported by both; our data suggest at a minimum that in IB objective measures do not neatly line up with subjective measures.)

We hope this addresses the referee’s concern that we dismiss the “the comparison of IB and blindsight too quickly”. We do not intend to dismiss that comparison at all, indeed we raise it because we consider it a serious hypothesis. Our aim is simply to raise one possible consideration against it. But, again, our main claim is quite consistent with sensitivity in IB being akin to “blindsight”.

We also agree with the referee that a possible explanation of why some subjects say they do not notice something unusual in IB paradigms, is not because they didn’t notice anything but because they didn’t consider the unexpected stimulus sufficiently unusual. However, the reviewer is incorrect that we did not mention this interpretation; to the contrary, it was precisely the kind of concern which led us to be dissatisfied with standard IB methods and so motivated our approach. As we wrote in our main text: “However, yes/no questions of this sort are inherently and notoriously subject to bias… For example, observers might be under-confident whether they saw anything (or whether what they saw counted as unusual); this might lead them to respond “no” out of an excess of caution.” On our view, this is exactly the kind of reason (among other reasons) that one cannot rely on yes/no reports of noticing unusual stimuli, even though the field has relied on just these sorts of questions in just this way.

We do not, however, think that this explanation accounts for why all subjects fail to report noticing, nor do we think that it accounts for our finding of above-chance sensitivity amongst non-noticers. This is for two critical reasons. First, whereas the word “unusual” did appear in the yes/no question in our Experiments 1-3, it did not appear in our Experiments 4 and 5 on dynamic IB. (In both cases, we used the exact wording of such questions in the experiments we were basing our work on.) And, of course, we still found significant residual sensitivity amongst non-noticers in Experiments 4 and 5. Second, in relation to our confidence experiment, we think it unlikely that subjects who were highly confident that they did not notice anything unusual only said that because they thought what they had seen was insufficiently unusual. Yet even in this group of subjects who were maximally confident that they did not notice anything unusual, we still found residual sensitivity.

(7) The authors use sub-optimal questioning procedures to challenge the existence of the phenomenon this questioning is intended to demonstrate. A more neutral interpretation of this study is that it is a critique on methods in IB research, not a critique on IB as a manipulation or phenomenon. The authors neglect to mention the dozens of modern IB experiments that have improved upon the simple Y/N IB questioning methods. For example, in Michael Cohen's IB experiments (e.g., Cohen et al., 2011; Cohen et al., 2020; Cohen et al., 2021), he uses a carefully crafted set of probing questions to conservatively ensure that subjects who happened to notice the critical stimuli have every possible opportunity to report seeing them. In other experiments (e.g., Hirschhorn et al., 2024; Pitts et al., 2012), researchers not only ask the Y/N question but then follow this up by presenting examples of the critical stimuli so subjects can see exactly what they are being asked about (recognition-style instead of free recall, which is more sensitive). These follow-up questions include foil stimuli that were never presented (similar to the stimulus-absent trials here), and ask for confidence ratings of all stimuli. Conservative, pre-defined exclusion criteria are employed to improve the accuracy of their IB-rate estimates. In these and other studies, researchers are very cautious about trusting what subjects report seeing, and in all cases, still find substantial IB rates, even to highly salient stimuli. The authors should consider at least mentioning these improved methods, and perhaps consider using some of them in their future experiments.

The concern that we do not sufficiently discuss the range of “improved” methods in IB studies is well-taken. A similar concern is raised by Reviewer #2 (Dr. Cohen). To address the concern, we have added to our manuscript a substantial new discussion of such improved methods. However, although we do agree that these methods can be helpful and may well address some of the methodological concerns which our paper raises, we do not think that they are a panacea. Thus, our discussion of these methods also includes a substantial discussion of the problems and pitfalls with such methods which led us to favor our own simple forced-response and 2afc questions, combined with SDT analysis. We think this approach is superior both to the classic approach in IB studies *and* to the approach raised by the reviewers.

In particular, we have four main concerns about the follow up questions now commonly used in the field:

First, many follow up questions are used not to exclude people from the IB group but to *include* people in the IB group. Thus, Most et al. 2001 asked follow up questions but used these to increase their IB group, only excluding subjects from the IB group if they *both* reported seeing and answered their follow ups incorrectly: “Observers were regarded as having seen the unexpected object if they answered 'yes' when asked if they had seen anything on the critical trial that had not been present before and if they were able to describe its color, motion, or shape." This means that subjects who saw the object but failed to see its color, say, would be treated as inattentionally blind. This has the purpose of inflating IB rates, in exactly the way our paper is intended to critique. So, in our view this isn’t an improvement but rather part of the approach we take issue with.

Second, many follow up questions remain yes/no questions or nearby variants, all of which are subject to response bias. For example, in Cohen’s studies which the reviewer mentions, it is certainly true that “he uses a carefully crafted set of probing questions to conservatively ensure that subjects who happened to notice the critical stimuli have every possible opportunity to report seeing them.” We agree that this improves over a simple yes/no question in some ways. However, such follow up probes nonetheless remain yes/no questions, subject to response bias, e.g.:

(1) “Did you notice anything strange or different about that last trial?”

(2) “If I were to tell you that we did something odd on the last trial, would you have a guess as to what we did?”

(3) “If I were to tell you we did something different in the second half of the last trial, would you have a guess as to what we did?”

(4) “Did you notice anything different about the colors in the last scene?”

Indeed, follow up questions of this kind can be especially susceptible to bias, since subjects may be reluctant to “take back” their earlier answers and so be conservative in responding positively to avoid inconsistency or acknowledgement of earlier error. This may explain why such follow up questions produce remarkable consistency despite their rather different wording. Thus, Simons and Chabris (1999) report: “Although we asked a series of questions escalating in specificity to determine whether observers had noticed the unexpected event, only one observer who failed to report the event in response to the first question (“did you notice anything unusual?'') reported the event in response to any of the next three questions (which culminated in “did you see a ... walk across the screen?''). Thus, since the responses were nearly always consistent across all four questions, we will present the results in terms of overall rates of noticing.” Thus, while there are undoubtedly merits to these follow ups, they do not resolve problems of bias.

This same basic issue affects the follow up question used in Pitts et al. 2012 which the reviewer mentions. Pitts et al. write: “If a participant reported not seeing any patterns and rated their confidence in seeing the square pattern (once shown the sample) as a 3 or less (1 = least confident, 5 = most confident), she or he was placed in Group 1 and was considered to be inattentionally blind to the square patterns.” The confidence rating follow-up question here remains subject to bias. Moreover, and strikingly, the inclusion criterion used means that subjects who were moderately confident that they saw the square pattern when shown (i.e. answered 3) were counted as inattentionally blind (!). We do not think this is an appropriate inclusion criterion.

The third problem is that follow up questions are often free/open-response. For instance, Most et al. (2005) ask the follow up question: "If you did see something on the last trial that had not been present during the first two trials, what color was it? If you did not see something, please guess." This is a much more difficult and to that extent less sensitive question than our binary forced-response/2afc questions. For this reason, we believe our follow up questions are more suitable for ascertaining low levels of sensitivity.

The fourth and final issue is that whereas 2afc questions are criterion free (in that they naturally have an unbiased decision rule), this is in fact not true of _n_afc questions in general, nor is it true in general of *delayed n-alternative match to sample* designs. Thus, even when limited response options are given, they are not immune to response biases and so require SDT analysis. Moreover, some such tasks can involve decision spaces which are often poorly understood or difficult to analyze without making substantial assumptions about observer strategy.

This last point (as well as the first) is relevant to Hirshhorn et al. 2024. Hirshhorn et al. write that they “used two awareness measures. Firstly, participants were asked to rate stimulus visibility on the Perceptual Awareness Scale (PAS, a subjective measure of awareness: Ramsøy & Overgaard, 2004), and then they were asked to select the stimulus image from an array of four images (an objective measure: Jakel & Wichmann, 2006).”

While certainly an improvement on simple yes/no questioning, the PAS remains subject to response bias. On the other hand, we applaud Hirshhorn et al.’s use of objective measures in the context of IB which of course our design implements. However, while Hirshhorn et al. 2024 suggest that their task is a spatial 4afc following the recommendation of this design by Jakel & Wichmann (2006), it is strictly a 4-alternative delayed match to sample task, so it is doubtful if it can be considered a preferred psychophysical task for the reasons Jakel & Wichmann offer. Regardless, the more crucial point is that observers in such a task might be biased towards one alternative as opposed to another. Thus, use of *d′* (as opposed to percent correct as in Hirshhorn et al. 2024) is crucial in assessing performance in such tasks.

For all these reasons, then, while we agree that the field has taken significant steps to move beyond the simple yes/no question traditionally used in IB studies (and we have revised our manuscript to make this clear); we do not think it has resolved the methodological issues which our paper seeks to highlight and address, and we believe that our approach contributes something additional that is not yet present in the literature. We have now revised our manuscript to make these points much more clearly, and we thank the reviewer for prompting these improvements.

**Reviewer #2 (Public review):**

In this study, Nartker et al. examine how much observers are conscious of using variations of classic inattentional blindness studies. The key idea is that rather than simply asking observers if they noticed a critical object with one yes/no question, the authors also ask follow-up questions to determine if observers are aware of more than the yes/no questions suggest. Specifically, by having observers make forced choice guesses about the critical object, the authors find that many observers who initially said "no" they did not see the object can still "guess" above chance about the critical object's location, color, etc. Thus, the authors claim, that prior claims of inattentional blindness are mistaken and that using such simple methods has led numerous researchers to overestimate how little observers see in the world. To quote the authors themselves, these results imply that "inattentionally blind subjects consciously perceive these stimuli after all... they show sensitivity to IB stimuli because they can see them."

Before getting to a few issues I have with the paper, I do want to make sure to explicitly compliment the researchers for many aspects of their work. Getting massive amounts of data, using signal detection measures, and the novel use of a "super subject" are all important contributions to the literature that I hope are employed more in the future.

We really appreciate this comment and that the reviewer found our work to make these important contributions to the literature. We wrote this paper expecting not everyone to accept our conclusions, but hoping that readers would see the work as making a valuable contribution to the literature promoting an underexplored alternative in a compelling way. Given that this reviewer goes on to express some skepticism about our claims, it is especially encouraging to see this positive feedback up top!

Main point 1: My primary issue with this work is that I believe the authors are misrepresenting the way people often perform inattentional blindness studies. In effect, the authors are saying, "People do the studies 'incorrectly' and report that people see very little. We perform the studies 'correctly' and report that people see much more than previously thought." But the way previous studies are conducted is not accurately described in this paper. The authors describe previous studies as follows on page 3:

"Crucially, however, this interpretation of IB and the many implications that follow from it rest on a measure that psychophysics has long recognized to be problematic: simply asking participants whether they noticed anything unusual. In IB studies, awareness of the unexpected stimulus (the novel shape, the parading gorilla, etc.) is retroactively probed with a yes/no question, standardly, "Did you notice anything unusual on the last trial which wasn't there on previous trials?". Any subject who answers "no" is assumed not to have any awareness of the unexpected stimulus.

If this quote were true, the authors would have a point. Unfortunately, I do not believe it is true. This is simply not how many inattentional blindness studies are run. Some of the most famous studies in the inattentional blindness literature do not simply as observes a yes/no question e.g., the invisible gorilla (Simons et al. 1999), the classic door study where the person changes (Simons and Levin, 1998), the study where observers do not notice a fight happening a few feet from them (Chabris et al., 2011). Instead, these papers consistently ask a series of follow-up questions and even tell the observers what just occurred to confirm that observers did not notice that critical event (e.g., "If I were to tell you we just did XYZ, did you notice that?"). In fact, after a brief search on Google Scholar, I was able to relatively quickly find over a dozen papers that do not just use a yes/no procedure, and instead as a series of multiple questions to determine if someone is inattentionally blind. In no particular order some papers (full disclosure: including my own):

(1) Most et al. (2005) Psych Review

(2) Drew et al. (2013) Psych Science

(3) Drew et al. (2016) Journal of Vision

(4) Simons et al. (1999) Perception

(5) Simons and Levin (1998) Perception

(6) Chabris et al. (2011) iPerception

(7) Ward & Scholl (2015) Psych Bulletin and Review

(8) Most et al. (2001) Psych Science

(9) Todd & Marois (2005) Psych Science

(10) Fougnie & Marois (2007) Psych Bulletin and Review

(11) New and German (2015) Evolution and Human Behaviour

(12) Jackson-Nielsen (2017) Consciousness and cognition

(13) Mack et al. (2016) Consciousness and cognition

(14) Devue et al. (2009) Perception

(15) Memmert (2014) Cognitive Development

(16) Moore & Egeth (1997) JEP:HPP

(17) Cohen et al. (2020) Proc Natl Acad Sci

(18) Cohen et al. (2011) Psych Science

This is a critical point. The authors' key idea is that when you ask more than just a simple yes/no question, you find that other studies have overestimated the effects of inattentional blindness. But none of the studies listed above only asked simple yes/no questions. Thus, I believe the authors are mis-representing the field. Moreover, many of the studies that do much more than ask a simple yes/no question are cited by the authors themselves! Furthermore, as far as I can tell, the authors believe that if researchers do these extra steps and ask more follow-ups, then the results are valid. But since so many of these prior studies do those extra steps, I am not exactly sure what is being criticized.

To make sure this point is clear, I'd like to use a paper of mine as an example. In this study (Cohen et al., 2020, Proc Natl Acad Sci USA) we used gaze-contingent virtual reality to examine how much color people see in the world. On the critical trial, the part of the scene they fixated on was in color, but the periphery was entirely in black and white. As soon as the trial ended, we asked participants a series of questions to determine what they noticed. The list of questions included:

(1) "Did you notice anything strange or different about that last trial?"

(2) "If I were to tell you that we did something odd on the last trial, would you have a guess as to what we did?"

(3) "If I were to tell you we did something different in the second half of the last trial, would you have a guess as to what we did?"

(4) "Did you notice anything different about the colors in the last scene?"

(5) We then showed observers the previous trial again and drew their attention to the effect and confirmed that they did not notice that previously.

In a situation like this, when the observers are asked so many questions, do the authors believe that "the inattentionally blind can see after all?" I believe they would not say that and the reason they would not say that is because of the follow-up questions after the initial yes/no question. But since so many previous studies use similar follow-up questions, I do not think you can state that the field is broadly overestimating inattentional blindness. This is why it seems to me to be a bit of a strawman: most people do not just use the yes/no method.

We appreciate this reviewer raising this issue. As he (Dr. Cohen) states, his “primary issue” concerns our discussion of the broader literature (which he worries understates recent improvements made to the IB methodology), rather than, e.g., the experiments we’ve run. We take this concern very seriously and address it comprehensively here.

A very similar issue is identified by Reviewer #1, comment (7). To review some of what we say in reply to them: To address the concern we have added to our manuscript a substantial new discussion of such improved methods. However, although we do agree that these methods can be helpful and may well address some of the methodological concerns which our paper raises, we do not think that they are a panacea. Thus, our discussion of these methods also includes a substantial discussion of the problems and pitfalls with such methods which led us to favor our own simple forced-response and 2afc questions, combined with SDT analysis. We think this approach is superior both to the classic approach in IB studies *and* to the approach raised by the reviewers.

In particular, we have three main concerns about the follow up questions now commonly used in the field:

First, many follow up questions are used not to *exclude* subjects from the IB group but to *include* subjects in the IB group. Thus, Most et al. (2001) asked follow up questions but used these to increase their IB group, only excluding subjects from the IB group if they *both* reported seeing and failed to answer their follow ups correctly: “Observers were regarded as having seen the unexpected object if they answered 'yes' when asked if they had seen anything on the critical trial that had not been present before and if they were able to describe its color, motion, or shape." This means that subjects who saw the object but failed to describe it in these respects would be treated as inattentionally blind. This is problematic since failure to describe a feature (e.g., color, shape) does not imply a complete lack of information concerning that feature; and even if a subject did lack all information concerning these features of an object, this would not imply a complete failure to see the object. Similarly, Pitts et al. (2012) asked subjects to rate their confidence in their initial yes/no response from 1 = least confident to 5 = most confident, and used these ratings to include in the IB group those who rated their confidence in seeing at 3 or less. This is evidently problematic, since there is a large gap between being under confident that one saw something and being completely blind to it. More generally, using follows up to inflate IB rates in such ways raises precisely the kinds of issues our paper is intended to critique. So in our view this isn’t an improvement but rather part of the approach we take issue with.

Second, many follow up questions remain yes/no questions or nearby variants, all of which are subject to response bias. For example, in the reviewer’s own studies (Cohen et al. 2020, 2011; see also: Simons et al., 1999; Most et al., 2001, 2005; Drew et al., 2013; Memmert, 2014) a series of follow up questions are used to try and ensure that subjects who noticed the critical stimuli are given the maximum opportunity to report doing so, e.g.:

(1) “Did you notice anything strange or different about that last trial?”

(2) “If I were to tell you that we did something odd on the last trial, would you have a guess as to what we did?”

(3) “If I were to tell you we did something different in the second half of the last trial, would you have a guess as to what we did?”

(4) “Did you notice anything different about the colors in the last scene?”

We certainly agree that such follow up questions improve over a simple yes/no question in some ways. However, such follow up probes nonetheless remain yes/no questions, intrinsically subject to response bias. Indeed, follow up questions of this kind can be especially susceptible to bias, since subjects may be reluctant to “take back” their earlier answers and so be conservative in responding positively to avoid inconsistency or acknowledgement of earlier error. This may explain why such follow up questions produce remarkable consistency despite their rather different wording. Thus, Simons and Chabris (1999) report: “Although we asked a series of questions escalating in specificity to determine whether observers had noticed the unexpected event, only one observer who failed to report the event in response to the first question (“did you notice anything unusual?'') reported the event in response to any of the next three questions (which culminated in “did you see a ... walk across the screen?''). Thus, since the responses were nearly always consistent across all four questions, we will present the results in terms of overall rates of noticing.” Thus, while there are undoubtedly merits to these follow ups, they do not resolve problems of bias.

It is also important to recognize that whereas 2afc questions are criterion free (in that they naturally have an unbiased decision rule), this is not true of _n_afc nor delayed *n*-alternative match to sample designs in general. Performance in such tasks thus requires SDT analysis – which itself may be problematic if the decision space is not properly understood or requires making substantial assumptions about observer strategy.

Third, and finally, many follow up questions are insufficiently sensitive (especially with small sample sizes). For instance, Todd, Fougnie & Marois (2005) used a 12-alternative match-tosample task (see similarly: Fougnie & Marois, 2007; Devue et al., 2009). And Most et al. (2005) asked an open-response follow-up: “If you did see something on the last trial that had not been present during the first two trials, what color was it? If you did not see something, please guess.” These questions are more difficult and to that extent less sensitive than binary forced-response/2afc questions of the sort we use in our own studies – a difference which may be critical in uncovering degraded perceptual sensitivity.

For all these reasons, then, while we agree that the field has taken significant steps to move beyond the simple yes/no question traditionally used in IB studies (and we have revised our manuscript to make this clear); we do not think it has resolved the methodological issues which our paper seeks to highlight and address, and we believe that our approach of using 2afc or forced-response questions combined with signal detection analysis is an important improvement on prior methods and contributes something additional that is not yet present in the literature. We have now revised our manuscript to make these points much clearer.

Other studies that improve on the standard methodology

This reviewer adds something else, however: A very helpful list of 18 papers which include follow ups and that he believes overcome many of the issues we raise in our paper. To just state our reaction bluntly: We are familiar with every one of these papers (indeed, one of them is a paper by one of us!), and while we think these are all very valuable contributions to the literature, it is our view that *none* of these 18 papers resolves the worries that led us to conduct our work.

Here we briefly comment on the relevant pitfalls in each case. We hope this serves to underscore the importance of our methodological approach.

(1) Most et al. (2005) *Psych Review*

Either a 2-item or 5-item questionnaire was used. The 2-item questionnaire ran as follows:

(1) On the last trial, did you see anything other than the 4 circles and the 4 squares (anything that had not been present on the original two trials)? Yes No

(2) If you did see something on the last trial that had not been present during the original two trials, please describe it in as much detail as possible.

This clearly does not substantially improve on the traditional simple yes/no question. Moreover, the second question (as well as being open-ended) was used to *include* additional subjects in the IB group, in that participants were counted as having seen the object only if they responded “yes” to Q1 *and in addition* “were able to report at least one accurate detail” in response to Q2. In other words, either a subject says “no” (and is treated as unaware), or says “yes” and then is asked to prove their awareness, as it were. If anything, this intensifies the concerns we raise, by inflating IB rates.

The 5-item questionnaire looked like this:

(1) On the last trial, did you see anything other than the black and white L’s and T’s (anything that had not been present on the first two trials)?

(2) If you did see something on the last trial that had not been present during the first two trials, please describe it.

(3) If you did see something on the last trial that had not been present during the first two trials, what color was it? If you did not see something, please guess. (Please indicate whether you did see something or are guessing)

(4) If you did see something during the last trial that had not been present in the first two trials, please draw an arrow on the “screen” below showing the direction in which it was moving. If you did not see something, please guess. (Please indicate whether you did see something or are guessing)

(5) If you did see something during the last trial that had not been present during the first two trials, please circle the shape of the object below [4 shapes are presented to choose from]. If you did not see anything, please guess. (Please indicate whether you did see something or are guessing)

Q5 was not used for analysis purposes. (It suffers from the second issue raised above.) Q1 is the traditional y/n question. Qs 2&3 are open ended. It is unclear how responses to Q4 were analyzed (at the limit it could be considered a helpful, forced-choice question – though it again would suffer from the second issue raised above). However, as noted with respect to the 2-item questionnaire, these responses were not used to exclude people from the IB group but to *include* people in it. So again, this approach does not in any way address the issues we are concerned about, and if anything, only makes them worse.

(2) Drew et al. (2013) *Psych Science*

All follow ups were yes/no: “we asked a series of questions to determine whether they noticed the gorilla: ‘Did the final trial seem any different than any of the other trials?’, ‘Did you notice anything unusual on the final trial?’, and, finally, ‘Did you see a gorilla on the final trial?’”. So, this paper essentially implements the standard methodology we mention (and criticize).

(3) Drew et al. (2016) *Journal of Vision*

Follow up questions were used, but the reported procedure does not provide sufficient details to evaluate them (we are only told: “After the final trial, they were asked: ‘On that last trial of the task, did you notice anything that was not there on previous trials?’ They then answered questions about the features of the unexpected stimulus on a separate screen (color, shape, movement, and direction of movement).”). It is not clear that these follow ups were used to exclude any subjects from the analysis. Finally, given that the unexpected object could be the same color as the targets/distractors, it is clear that biases would have been introduced which would need to be considered (but which were not).

(4) Simons & Chabris (1999) *Perception*

All follow ups were yes/no: “observers were … asked to provide answers to a surprise series of additional questions. (i) While you were doing the counting, did you notice anything unusual on the video? (ii) Did you notice any- thing other than the six players? (iii) Did you see anyone else (besides the six players) appear on the video? (iv) Did you see a gorilla [woman carrying an umbrella] walk across the screen? After any “yes'' response, observers were asked to provide details of what they noticed. If at any point an observer mentioned the unexpected event, the remaining questions were skipped.” As noted previously, the analyses in fact did not use these questions to exclude subjects since answers were so consistent.

(5) Simons and Levin (1998) *Perception*

This is a change detection paradigm, not a study of inattentional blindness. And in any case, one yes/no follow up was used: “Did you notice that I'm not the same person who approached you to ask for directions?”

(6) Chabris et al. (2011) *iPerception*

Two yes/no questions were asked: “we asked whether the subjects had seen anything unusual along the route, and then whether they had seen anyone fighting.” It seems that follow up questions (a request to describe the fight) were asked *only of those who said yes*.

This is in fact a common procedure – follow up questions only being asked of the “yes” group. As discussed, it is sometimes used to *increase* rates of IB, *compounding* the problem we identify in our paper. So this is another example of a follow-up question that makes the problem we identify worse, not better.

(7) Ward & Scholl (2015) Psych Bulletin and Review

Two yes/no questions were used: “...observers were asked whether they noticed ‘anything … that was different from the first three trials’ — and if so, to describe what was different. They were then shown the gray cross and asked if they had noticed it—and if so, to describe where it was and how it moved. Only observers who explicitly reported not noticing the cross were counted as ‘nonnoticers’ to be included in the final sample (N = 100).” In each case, combining the traditional noticing question with a request to describe and identify may have induced conservative response biases in the noticing question, since a subject might consider being able to describe or identify the unexpected stimulus a precondition of giving a positive answer to the noticing question.

(8) Most et al. (2001) *Psych Science*

The same 5-item questionnaire discussed above in relation to Most et al. (2005) was used:

(1) On the last trial, did you see anything other than the black and white L’s and T’s (anything that had not been present on the first two trials)?

(2) If you did see something on the last trial that had not been present during the first two trials, please describe it.

(3) If you did see something on the last trial that had not been present during the first two trials, what color was it? If you did not see something, please guess. (Please indicate whether you did see something or are guessing)

(4) If you did see something during the last trial that had not been present in the first two trials, please draw an arrow on the “screen” below showing the direction in which it was moving. If you did not see something, please guess. (Please indicate whether you did see something or are guessing)

(5) If you did see something during the last trial that had not been present during the first two trials, please circle the shape of the object below [4 shapes are presented to choose from]. If you did not see anything, please guess. (Please indicate whether you did see something or are guessing)

Q5 was not used for analysis purposes. (It suffers from the second issue raised above.) Q1 is the traditional yes/no question. Qs 2&3 are open ended. It is unclear how responses to Q4 were analyzed (at the limit it could be considered a helpful, forced-choice question – though it again would suffer from the second issue raised above). However, as noted with respect to the two item questionnaire in Most et al. 2005, these responses were not used to exclude people from the IB group but to *include* people in it. So again this approach does not in any way address the issues we are concerned about, and if anything only makes them worse.

(9) Todd, Fougnie & Marois (2005) *Psych Science*

“participants were probed with three questions to determine whether they had detected the critical stimulus ... .The first question assessed whether subjects had seen anything unusual during the trial; they responded ‘‘yes’’ or ‘‘no’’ by pressing the appropriate key on the keyboard. The second question asked participants to select which stimulus they might have seen among 12 possible objects and symbols selected from MacIntosh font databases. The third question asked participants to select the quadrant in which the critical stimulus may have appeared by pressing one of four keys, each of which corresponded to one of the quadrants.”

These follow ups were used to *include* people in the IB group: “In keeping with previous studies (Most et al., 2001), participants were considered to have detected the critical stimulus successfully if they (a) reported seeing an unexpected stimulus *and* (b) correctly selected its quadrant location.” In line with our third point about sensitivity, the object identity test transpired to be “too difficult even under full-attention conditions … Thus, performance with this question was not analyzed further.”

(10) Fougnie & Marois (2007) *Psych Bulletin and Review*

Same exact methods and problems as with Todd & Marois (2005) *Psych Science*, just discussed.

(11) New and German (2015) Evolution and Human Behaviour

“After the fourth trial containing the additional experimental stimulus, the participant was asked, “Did you see anything in addition to the cross on that trial?” and which quadrant the additional stimulus appeared in. They were then asked to identify the stimulus in an array which in Experiment 1 included two variants chosen randomly from the spider stimuli and the two needle stimuli. Participants in Experiment 2 picked from all eight stimuli used in that experiment.”

Our second concern about response biases and the need for appropriate SDT analysis of the 4/8 alternative tasks applies to all these questions. We also note that analyses were only performed on groups separately (those who detected/failed to detect, those who located/failed to locate, and those who identified/failed to identify) and on the group which did all three/failed to do any one of the three. Especially in light of the fact that some subjects could clearly detect the stimulus without being able to identity it (e.g.), the most stringent test given our concerns (which were not obviously New and German’s comparative concerns), would be to consider the group which could not detect, identify *or* localize.

(12) Jackson-Nielsen (2017) *Consciousness and cognition*

This is a very interesting example of a follow-up which used a 3-AFC recognition test:

“participants were immediately asked, ‘‘which display looks most like what you just saw?’ from 3 alternatives”. However, though such an objective test is definitely to be preferred in our view to an open-ended series of probes, the 3-AFC test administered clearly had issues with response biases, as discussed, and actually yielded significantly *below* chance performance in one of the experiments.

(13) Mack et al. (2016) Consciousness and cognition

The follow ups here were essentially yes/no combined with an assessment of surprise. Participants were asked to enter letters into a box, and if they did so “were immediately asked by the experimenter whether they had noticed anything different about the array on this last trial and if they did not, they were told that there had been no letters and their responses to that news were recorded. Clearly, if they expressed surprise, this would be compelling evidence that they were unaware of the absence of the letters. Those observers who did not enter letters and realized there were no letters present were considered aware of the absence.” So, this again has all of the same problems we identify, considering subjects unaware because they expressed surprise.

(14) Devue et al. (2009) *Perception*

An 8-alternative task was used. The authors were primarily interested in a comparative analysis and so did not use this task to exclude subjects. We note that an 8 alternative task is very demanding – compare the 12-alternative task used in Todd, Fougnie & Marois (2005). There was an attempt to investigate biases in a separate bias trial, however SDT measures were not used.

(15) Memmert (2014) Cognitive Development

“After watching the video and stating the number of passes, participants answered four questions (following Simons & Chabris, 1999): (1) While you were counting, did you perceive anything unusual on the video? (2) Did you perceive anything other than the six players? (3) Did you see anyone else (besides the six players) appear on the video? (4) Did you notice a gorilla walk across the screen? After any “yes” reply, children were asked to provide details of what they noticed. If at any point a child mentioned the unexpected event, the remaining questions were omitted.” All of these follow-up questions are yes/no judgments, used to determine awareness in exactly the way we critique as problematic.

(16) Moore & Egeth (1997) *JEP:HPP*

This study (which includes one of us, Egeth, as author) did use forced choice questions. In one case, the question was 2-alternative, in the other it was 4-alternative. In the latter case, SDT would have been appropriate but was not used. In the former case, it may have been that a larger sample would have revealed evidence of sensitivity to the background pattern (as it stood 55% answered the 2-alternative question correctly). Although these results have been replicated, unfortunately the replication in Wood and Simons 2019 used a 6-alternative recognition task and this was not analyzed using SDT. We also note that the task is rather difficult in this study. Wood and Simons report: “Exclusion rates were much higher than anticipated, primarily due to exclusions when subjects failed to correctly report the pattern on the full-attention trial; we excluded 361 subjects, or 58% of our sample.”

(17) Cohen et al. (2020) *Proc Natl Acad Sci*

While this paper improves over a simple yes/no question in some ways, especially in that it used the follow up questions to *exclude* subjects from the unaware (IB) group, the follow up probes nonetheless remain yes/no questions, subject to response bias, e.g.:

(1) “Did you notice anything strange or different about that last trial?”

(2) “If I were to tell you that we did something odd on the last trial, would you have a guess as to what we did?”

(3) “If I were to tell you we did something different in the second half of the last trial, would you have a guess as to what we did?”

(4) “Did you notice anything different about the colors in the last scene?”

Follow up questions of this kind can be especially susceptible to bias, since subjects may be reluctant to “take back” their earlier answers and so be conservative in responding positively to avoid inconsistency or acknowledgement of earlier error. This may explain why such follow up questions can produce remarkable consistency despite their rather different wording.

(18) Cohen et al. (2011) *Psych Science*

Here are the probes used in this study:

(1) Did you notice anything different on that trial?

(2) Did you notice something different about the background stream of images?

(3) Did you notice that a different type of image was presented in the background that was unique in some particular way?

(4) Did you see an actual photograph of a natural scene in that stream?

(5) If I were to tell you that there was a photograph in that stream, can you tell me what it was a photograph of?

Qs 1-4 are yes/no. Q5 is yes/no with an open-ended response. After this, a 5 or 6-alternative recognition test was administered. So again, this faces the same issues, since y/n questions are subject to bias in the way we have described, and many-alternative tests are more problematic than 2afc tests.

In summary

We *really* appreciate the care that went into compiling this list, and we agree that these papers and the improved methods they contain are relevant. But as hopefully made clear above, the approaches in each of these papers simply don’t solve the foundational issues our critique is aimed at (though they may address other issues). This is why we felt our new approach was necessary. And we continue to feel this way even after reading and incorporating these comments from Dr. Cohen.

Nevertheless, there is clearly lots for us to do in light of these comments. And so as noted earlier we have now added a very substantial new section to our discussion section to more fairly and completely portray the state of the art in this literature. This is really to our benefit in the end, since we now not only better acknowledge the diverse approaches present, but also set up ourselves to make our novel contribution exceedingly clear.

Main point 2: Let's imagine for a second that every study did just ask a yes/no question and then would stop. So, the criticism the authors are bringing up is valid (even though I believe it is not). I am not entirely sure that above chance performance on a forced choice task proves that the inattentionally blind can see after all. Could it just be a form of subliminal priming? Could there be a significant number of participants who basically would say something like, "No I did not see anything, and I feel like I am just guessing, but if you want me to say whether the thing was to the left or right, I will just 100% guess"? I know the literature on priming from things like change and inattentional blindness is a bit unclear, but this seems like maybe what is going on. In fact, maybe the authors are getting some of the best priming from inattentional blindness because of their large sample size, which previous studies do not use.

I'm curious how the authors would relate their studies to masked priming. In masked priming studies, observers say the did not see the target (like in this study) but still are above chance when forced to guess (like in this study). Do the researchers here think that that is evidence of "masked stimuli are truly seen" even if a participant openly says they are guessing?

We’re grateful to the reviewer for raising this question. As we say in response to Reviewer #1, our primary ambition in the paper is to establish, as our title suggests, residual sensitivity in IB. The ambition is quite neutral as to whether the sensitivity reflects conscious or unconscious processing (i.e. is akin to blindsight as traditionally conceived, or what the reviewer here suggests may be happening in masked priming). Since we were evidently insufficiently clear about this we have revised our manuscript in several places to clarify that we take our data primarily to support the more modest claim that there is residual sensitivity (conscious or unconscious) in the group of subjects who are traditionally classified as inattentionally blind. We believe that this claim has much more solid support in our data than our secondary and tentative suggestion about awareness.

This said, we do consider masked priming studies to be susceptible to the critique that performance may reflect degraded conscious awareness which is unreported because of conservative response criteria. There is good evidence that response criteria tend to be conservative near threshold (Björkman et al. 1993; see also: Railo et al. 2020), including specifically in masked priming studies (Sand 2016, cited in Phillips 2021). So, we consider it a perfectly reasonable hypothesis that subjects who say they feel they are guessing in fact have conscious access to a degraded signal which is insufficient to reach a conservative response criterion but nonetheless sufficient to perform above chance in 2afc detection. Of course, we appreciate that this hypothesis is controversial, so it is not one we argue for in our paper (though we are happy to share our feelings about it here).

Main point 3: My last question is about how the authors interpret a variety of inattentional blindness findings. Previous work has found that observers fail to notice a gorilla in a CT scan (Drew et al., 2013), a fight occurring right in front of them (Chabris et al., 2011), a plane on a runway that pilots crash into (Haines, 1991), and so forth. In a situation like this, do the authors believe that many participants are truly aware of these items but simply failed to answer a yes/no question correctly? For example, imagine the researchers made participants choose if the gorilla was in the left or right lung and some participants who initially said they did not notice the gorilla were still able to correctly say if it was in the left or right lung. Would the authors claim "that participant actually did see the gorilla in the lung"? I ask because it is difficult to understand what it means to be aware of something as salient as a gorilla in a CT scan, but say "no" you didn't notice it when asked a yes/no question. What does it mean to be aware of such important, ecologically relevant stimuli, but not act in response to them and openly say "no" you did not notice them?

Our view is that in such cases, observers may well have a “degraded” percept of the relevant feature (gorilla, plane, fight etc.). But crucially we do not suggest that this percept is sufficient for observers to recognize the object/event as a gorilla, plane, fight etc. Our claim is only that, in our studies at least, observers (as a group) do have enough information about the unexpected stimuli to locate them, and discriminate certain low level features better than chance. Crudely, it may be that subjects see the gorilla simply as a smudge or the plane as a shadowy patch etc. (One of us who is familiar with the gorilla CT scan stimuli notes that the gorilla is in fact rather hard to see even when you know which slide it is on, suggesting that they are not as “salient” as the reviewer suggests!)

More precisely, in the paper we write that in our view perhaps “...unattended stimuli are encoded in a partial or degraded way. Here we see a variety of promising options for future work to investigate. One is that unattended stimuli are only encoded as part of ensemble representations or summary scene statistics (Rosenholtz, 2011; Cohen et al., 2016). Another is that only certain basic “low-level” or “preattentive” features (see Wolfe & Utochkin, 2019 for discussion) can enter awareness without attention. A final possibility consistent with the present data is that observers can in principle be aware of individual objects and higher-level features under inattention but that the precision of the corresponding representations is severely reduced. Our central aim here is to provide evidence that awareness in inattentional blindness is not abolished. Further work is needed to characterize the exact nature of that awareness.” We hope this sheds light on our perspective while still being appropriately cautious not to go too far beyond our data.

Overall: I believe there are many aspects of this set of studies that are innovative and I hope the methods will be used more broadly in the literature. However, I believe the authors misrepresent the field and overstate what can be interpreted from their results. While I am sure there are cases where more nuanced questions might reveal inattentional blindness is somewhat overestimated, claims like "the inattentionally blind can see after all" or "Inattentionally blind subjects consciously perceive thest stimuli after all" seem to be incorrect (or at least not at all proven by this data).

Once again, we would like to thank this reviewer for his feedback, which obviously comes from a place of tremendous expertise on these issues. We appreciate his assessment that our studies are innovative and that our methodological advances will be of use more broadly. We also hear the reviewer loud and clear about the passages in question, which on reflection we agree are not as central to our case as the other claims we make (regarding residual sensitivity and conservative responding), and so we have now edited them accordingly to refocus our discussion on only those claims that are central and supported. Thank you for making our paper stronger!

**Reviewer #3 (Public review):**

Summary:

Authors try to challenge the mainstream scientific as well as popularly held view that Inattentional

Blindness (IB) signifies subjects having no conscious awareness of what they report not seeing (after being exposed to unexpected stimuli). They show that even when subjects indicate NOT having seen the unexpected stimulus, they are at above chance level for reporting features such as location, color or movement of these stimuli. Also, they show that 'not seen' responses are in part due to a conservative bias of subjects, i.e. they tend to say no more than yes, regardless of actual visibility. Their conclusion is that IB may not (always) be blindness, but possibly amnesia, uncertainty etc.

We just thought to say that we felt this was a very accurate summary of our claims, and in ways underscore the modesty we had hoped to convey. This is especially true of the reviewer’s final sentence: “Their conclusion is that IB may not (always) be blindness, but possibly amnesia, uncertainty etc.”; as we noted in response to other reviewers, our claim is not that IB doesn’t exist, that subjects are always conscious of the stimulus, etc.; it is only that the cohort of IB subjects show sensitivity to the unattended stimulus in ways that suggest they are not as blind as traditionally conceived. Thank you for reading us as intended!

Strengths:

A huge pool of (25.000) subjects is used. They perform several versions of the IB experiments, both with briefly presented stimuli (as the classic Mack and Rock paradigm), as well as with prolonged stimuli moving over the screen for 5 seconds (a bit like the famous gorilla version), and all these versions show similar results, pointing in the same direction: above chance detection of unseen features, as well as conservative bias towards saying not seen.

We’re delighted that the reviewer appreciated these strengths in our manuscript!

Weaknesses:

Results are all significant but effects are not very strong, typically a bit above chance. Also, it is unclear what to compare these effects to, as there are no control experiments showing what performance would have been in a dual task version where subjects have to also report features etc for stimuli that they know will appear in some trials

The backdrop to the experiments reported here is the “consensus view” (Noah & Mangun, 2020) according to which inattention completely abolishes perception, such that subjects undergoing IB “have no awareness at all of the stimulus object” (Rock et al., 1992) and that “one can have one’s eyes focused on an object or event … without seeing it at all” (Carruthers, 2015). In this context, we think our findings of significant above-chance sensitivity (e.g., *d′* = 0.51 for location in Experiment 1; chance, of course, would be *d′* = 0 here) are striking and constitute strong evidence against the consensus view. We of course agree that the residual sensitivity is far lower than amongst subjects who noticed the stimulus. For this reason, we certainly believe that inattention has a dramatic impact on perception. To that extent, our data speak in favor of a “middle ground” view on which inattention substantially degrades but crucially does not abolish perception/explicit encoding. We see this as an importantly neglected option in a literature which has overly focused on seen/not seen binaries (see our section ‘Visual awareness as graded’).

Regarding the absence of a control condition, we think those conditions wouldn’t have played the same role in our experiments as they typically play in other experiments. As Reviewer #1 comments, the main role of such trials in previous work has been to *exclude* from analysis subjects who failed to report the unexpected stimulus on the divided and/or full attention control trials. As Reviewer #1 points out, excluding such subjects would very likely have ‘helped’ us. However, the practice is controversial. Indeed, in a review of 128 experiments, White et al. 2018 argue that the practice has “problematic consequences” and “may lead researchers to understate the pervasiveness of inattentional blindness". Since we wanted to offer as simple and demanding a test of residual sensitivity in IB as possible, we thus decided not to use any exclusions, and for that reason decided not to include divided/full attention trials.

As recommended, we discuss this decision not to include divided/full attention trials and our logic for not doing so in the manuscript. As we explain, not having those conditions makes it more impressive, not less impressive, that we observed the results we in fact did — it makes our results more interpretable, not less interpretable, and so absence of such conditions from our manuscript should not (in our view) be considered any kind of weakness.

There are quite some studies showing that during IB, neural processing of visual stimuli continues up to high visual levels, for example, Vandenbroucke et al 2014 doi:10.1162/jocn_a_00530 showed preserved processing of perceptual inference (i.e. seeing a kanizsa illusion) during IB. Scholte et al 2006 doi: 10.1016/j.brainres.2005.10.051 showed preserved scene segmentation signals during IB. Compared to the strength of these neural signatures, the reported effects may be considered not all that surprising, or even weak.

We agree that such evidence of neural processing in IB is relevant to — and perhaps indeed consistent with — our picture, and we’re grateful to the reviewer for pointing out further studies along those lines. Previously, we mentioned a study from Pitts et al., 2012 in which, as we wrote, “unexpected line patterns have been found to elicit the same Nd1 ERP component in both noticers and inattentionally blind subjects (Pitts et al., 2012).” We have added references to both the studies which the reviewer mentions – as well as an additional relevant study – to our manuscript in this context. Thank you for the helpful addition.

We do however think that our studies are importantly different to this previous work. Our question is whether processing under IB yields representations which are available for explicit report and so would constitute clear evidence of seeing, and perhaps even conscious experience. As we discuss, evidence for this kind of processing remains wanting: “A handful of prior studies have explored the possibility that inattentionally blind subjects may retain some visual sensitivity to features of IB stimuli (e.g., Schnuerch et al., 2016; see also Kreitz et al., 2020, Nobre et al., 2020). However, a recent meta-analysis of this literature (Nobre et al., 2022) argues that such work is problematic along a number of dimensions, including underpowered samples and evidence of publication bias that, when corrected for, eliminates effects revealed by earlier approaches, concluding “that more evidence, particularly from well-powered pre-registered experiments, is needed before solid conclusions can be drawn regarding implicit processing during inattentional blindness” (Nobre et al., 2022).” Our paper is aimed at addressing this question which evidence of neural processing can only speak to indirectly.

**Recommendations for the authors:**

**Reviewer #1 (Recommendations for the authors):**

(1) Please report all of the data, especially the number of subjects in each experiment that answered Y/N and the numbers of subjects in each of the Y and N groups that guessed a feature correctly/incorrectly on the 2AFC tasks. And also the confidence ratings for the 2AFC task (for comparison with the confidence ratings on the Y/N questions).

We now report all this data in our (revised) Supplementary Materials. We agree that this information will be helpful to readers.

(2) Consider adding a control condition with partial attention (dual task) or full attention (single task) to estimate the rates of seeing the critical stimulus when it's expected.

This is the only recommendation we have chosen not to implement. The reason, as we explain in detail above (especially in response to Reviewer #1 comment 5), is that this would not in fact be a “control condition” *in our studies*, and indeed would only inflate the biases we are concerned with in our work. As the referee comments, the main role of such trials in previous work has been to *exclude* from analysis subjects who failed to report the unexpected stimulus on the divided and/or full attention control trials. And the practice is controversial: Indeed, in a review of 128 experiments, White et al. 2018 argue that the practice has “problematic consequences” and “may lead researchers to *understate* the pervasiveness of inattentional blindness" (emphasis added). So, our choice not to have such conditions ensures an *especially stringent test* of our central claim. Not having those conditions (and their accompanying exclusions) makes our results more interpretable, not less interpretable, and so the absence of such conditions from our manuscript should not (in our view) be considered any kind of weakness.

We have added a paragraph to our “Design and analytical approach” section explaining the logic behind our deliberate decision not to include divided or full attention trials in our experiments. (For even fuller discussion, see our response to Reviewer #1’s comment 5 above.)

(3) Consider revising the interpretations to be more precise about the distinction between the super subject being above chance versus each individual subject who cannot be at chance or above chance because there was only a single trial per subject.

We have now done this throughout the manuscript, as discussed above. We have also added a substantive additional discussion to our “Design and analytical approach” section discussing what should be said about individual subjects in light of our group level data.

This was a very helpful point, and greatly clarifies the claims we wish to make in the paper. Thank you for this comment, which has certainly made our paper stronger.

**Reviewer #2 (Recommendations for the authors):**

I would be curious to hear the authors' response to two points:

(1) What do they have to say about prior studies that do more than just ask yes/no questions (and ask several follow-ups)? Are those studies "valid"?

A very substantial new discussion of this important point has been added. As you will see above, we comment on every one of the 18 papers this reviewer raised (as well as the general argument made); we contend that while many of these papers improve on past methodology in various ways, most in fact do “just ask yes/no questions”, and none of them makes the methodological advance we offer in our manuscript. However, this discussion has helped us clarify that very advance, and so working through this issue has really helped us improve our paper and make its relation to existing literature that much clearer. Thank you for raising this crucial point.

(2) Do the authors think it is possible that in many cases, people are just guessing about a critical item's location or color and this is at least in part a form of priming?

We have clarified our discussion in numerous places to further emphasize that our main point concerns above-chance sensitivity, not awareness. Given this, we take very seriously the hypothesis that something like priming of a kind sometimes proposed to occur in cases of blindsight or other putative cases of unconscious perception could be what is driving the responses in non-noticers.

**Reviewer #3 (Recommendations for the authors):**

(1) Control dual task version with expected stimuli would be nice

We have added a paragraph to our “Design and analytical approach” section explaining the logic behind our deliberate decision not to include divided or full attention trials, which would not in fact be a “control” task in our experiments. For full discussion, see our response to Reviewer 3 above, as well as our summary here in the Recommendations for Authors section in responding to Reviewer 1, recommendation (2).

(2) Please do a better job in discussing and introducing experiments about neural signatures during IB.

A discussion of Vandenbroucke et al. 2014 and Scholte et al. 2006 has been added to our discussion of neural signatures in IB, as well as an additional reference to an important early study of semantic processing in IB (Rees et al., 1999). Thank you for these very helpful suggestions!
